# Prepulse inhibition deficit as a transdiagnostic process in neuropsychiatric disorders: a systematic review

**DOI:** 10.1186/s40359-023-01253-9

**Published:** 2023-08-07

**Authors:** Daniel Santos-Carrasco, Luis Gonzalo De la Casa

**Affiliations:** Department of Experimental Psychology, University of Seville, Seville, Spain

**Keywords:** Sensorimotor gating, Prepulse inhibition, Startle response, Neuropsychiatric disorders, Transdiagnostic process, Pre-attentional filtering

## Abstract

**Background:**

Psychopathological research is moving from a specific approach towards transdiagnosis through the analysis of processes that appear transversally to multiple pathologies. A phenomenon disrupted in several disorders is prepulse inhibition (PPI) of the startle response, in which startle to an intense sensory stimulus, or pulse, is reduced if a weak stimulus, or prepulse, is previously presented.

**Objective and methods:**

The present systematic review analyzed the role of PPI deficit as a possible transdiagnostic process for four main groups of neuropsychiatric disorders: (1) trauma-, stress-, and anxiety-related disorders (2) mood-related disorders, (3) neurocognitive disorders, and (4) other disorders such as obsessive-compulsive, tic-related, and substance use disorders. We used Web of Science, PubMed and PsycInfo databases to search for experimental case-control articles that were analyzed both qualitatively and based on their potential risk of bias. A total of 64 studies were included in this systematic review. Protocol was submitted prospectively to PROSPERO 04/30/2022 (CRD42022322031).

**Results and conclusion:**

The results showed a general PPI deficit in the diagnostic groups mentioned, with associated deficits in the dopaminergic neurotransmission system, several areas implied such as the medial prefrontal cortex or the amygdala, and related variables such as cognitive deficits and anxiety symptoms. It can be concluded that the PPI deficit appears across most of the neuropsychiatric disorders examined, and it could be considered as a relevant measure in translational research for the early detection of such disorders.

**Supplementary Information:**

The online version contains supplementary material available at 10.1186/s40359-023-01253-9.

## Introduction

The transdiagnostic model in psychopathology goes beyond the existing diagnostic categories, proposing a more representative classification system [1, 2]. Its origin lies in the existence of psychopathological processes that operate as common mechanisms in several disorders [3, 4]. This model contributes to the understanding of psychopathological comorbidity, and allows the generalization of knowledge between disorders [2]. It is also more efficient from an applied perspective since it enables the development of new treatments focused on common factors between disorders rather than specific interventions (e.g., Sakiris and Berle [5]).

Within the framework of this model, the National Institute of Mental Health (NIMH) created the Research Domain Criteria (RDoC) to shift the focus from diagnoses based on particular symptoms to the identification of common mechanisms from a cross-sectional perspective [6–8], thus allowing research into dimensional psychopathological classification systems [9, 10]. From these approaches derive some standards for transdiagnostic research [2] such as (1) assessing psychopathological processes in groups of patients with different disorders [11]; (2) evaluating and integrating knowledge from different levels of analysis [12]; and (3) dimensional proposals should specify the relationship between diagnosis-specific and transdiagnostic deficits [13].

Research on common mechanisms to multiple disorders has increased in recent years. Thus, processes such as psychological inflexibility [14], insomnia [15], intolerance of uncertainty [16], hypervigilance [17], perfectionism [18], rumination [19], and dissociation [20] have been proposed as transdiagnostic factors. Among them, cognitive deficits in the domains of selective attention and information filtering have gained much attention as a common factor in many different disorders [21, 22]. More specifically, sensory gating deficits such as P50 component suppression [23, 24] and sensorimotor deficits as the pre-pulse inhibition of the startle response [25] appear in a wide range of neuropsychiatric disorders [26]. In this review, we will focus on the second paradigm.

The startle response is a reflex behaviorally expressed as the contraction of certain muscles in response to the presentation of an intense and unexpected stimulus [25]. Such response prepares the organism to face potentially dangerous situations [27, 28]. In spite it is a reflex response, its intensity changes under different circumstances, such as, for example, habituation induced by repeated presentations of the startle-inducing stimulus (e.g., Pilz and Schnitzler [29]), or pre-pulse inhibition (e.g., Hoffman and Searle [30]; Graham [31]).

Pre-pulse Inhibition (PPI) is expressed as a decrease of the startle response to an intense stimulus (pulse) when it is preceded by a stimulus of lower intensity (prepulse) [30, 32]. The magnitude of PPI and startle response is usually assessed in humans by registering the electromyographic response of the orbicularis oculi muscle [33], using an experimental task in which two auditory tones (prepulse, and pulse) are presented with an inter-stimulus interval ranging from 30 to 500 milliseconds [34].

This measure has been proposed as an operational index of sensorimotor gating [35–38] since it integrates information from both sensory stimuli and motor responses [21]. Graham [31] proposed that two automatic processes are active when the weak stimulus (prepulse) precedes the intense one (pulse): One intended to process the prepulse, and the second one inhibiting pulse processing [39]. This hypothesis has received physiological support, since attention to a stimulus activates a brain inhibitory mechanism that blocks attention for an interval ranging between 30 and 500 ms [40].

From a neurobiological level, PPI involves the dopaminergic system, as well as the serotonergic, GABAergic, and glutamatergic systems of cortical and subcortical structures [41]. Specifically, an increase in dopaminergic activity reduces PPI [42, 43]. Similarly, the corticostriatal-pallidopontine circuit plays a crucial role in PPI due to efferent connections from different areas (prefrontal cortex, thalamus, hippocampus, amygdala, striatum, accumbens, and pallidum nuclei) to the pedunculopontine nucleus [44–46].

PPI has been suggested as a translational research tool [47, 48], since its deficit has been verified in different neuropsychiatric disorders with common neurobiological correlates [49, 50], and by the presence of affective components such as anxiety and stress [51, 52]. Specifically, the group where the most potent evidence of a PPI deficit is found is in schizophrenia spectrum disorders, where this deficit has been proposed as a biomarker [53, 54] and an endophenotype [55–57] of psychosis. This has been corroborated in a recent meta-analysis and in a systematic review, which found a widespread deficit of PPI in individuals within the schizophrenia spectrum [58], but not in their first-degree relatives [59], respectively.

On the other hand, PPI deficits have also been consistently found in psychopathological conditions close to the spectrum, such as patients with schizotypal personality disorder [60, 61]. Regarding the autism spectrum, a recent meta-analysis has revealed that the majority of patients with one of the spectrum disorders exhibit impaired PPI compared to controls, although this difference is more pronounced in children/adolescents than in adults [62]. Less consistency is found in other neurodevelopmental disorders [63], where the PPI deficit appears to be mediated by medication [64], as well as whether the PPI protocol instructed to attend to the pulses or not [65, 66].

Regarding the group of disorders related to trauma, stress, and anxiety, there is less consistency in the literature about a possible common deficit in sensorimotor gating. According to the fifth version of the Diagnostic and Statistical Manual of Mental Disorders (DSM-5) (American Psychiatric Association [67]), the main trauma- and stressor-related disorders are Post-Traumatic Stress Disorder (PTSD) and acute stress disorder, while anxiety disorders are mainly social anxiety disorder, panic disorder, and generalized anxiety disorder. With respect to PPI deficit, some contradictory results have been reported for panic disorder, PTSD, and social anxiety disorder [52, 68].

Discrepancies in PPI deficits have also been found in mood disorders, with bipolar disorder and major depressive disorder receiving more attention [68]. The depressive, bipolar, and related disorders group (DSM-5) is represented principally by major depressive disorder, persistent depressive disorder, bipolar disorder, bipolar disorder type II, and cyclothymic disorder [67].

Similarly, research on neurocognitive disorders has revealed some apparently contradictory results. This group mainly comprises Alzheimer’s, Parkinson’s, and Huntington’s disease, traumatic brain injury, stroke, HIV infection, and Lewy bodies [67]. Specifically, studies on PPI have been conducted in patients with Alzheimer’s, Parkinson’s, and Huntington’s diseases [21, 69]. In particular, a PPI deficit has been proposed as a biological marker for the differential diagnosis of mild cognitive impairment and Alzheimer’s disease [70]. In Parkinson’s and Huntington’s disease, evidence is scarce, although it also points to a possible PPI disruption [57].

Other disorders that have been associated with PPI deficits are highly varied. For example, in the case of obsessive-compulsive disorder (OCD), the deficit in patients varies depending on methodological differences among studies [68], as differences with controls or normal PPI ratios are found depending on the psychopharmacological status of the patients [71, 72]. However, it appears that the deficit in OCD is mediated by the prior presence of tics among patients [71], which makes sense considering the clear sensorimotor gating deficit in Gilles de la Tourette syndrome [34, 73, 74].

In addition to these disorders, considering that PPI represents an essential paradigm in the field of psychopharmacology [21, 36], it is relevant to discuss substance use disorders. Within this field, PPI has been primarily studied in relation to two substances: cannabis and stimulants. The results in this group of disorders seem to be contradictory, with an apparent deficit of PPI in the case of cannabis use but an improvement in this index with the use of stimulants [68]. However, these results need to be analyzed in detail, as they depend on multiple variables such as the chronicity of use, the stage of the disorder (e.g., abuse vs. relapse), or the paradigm used [68]. Additionally, substance misuse disorders present a high rate of comorbidity with the previously mentioned disorders (e.g., Alsuhaibani et al., 2021 [75]).

Based on available data on the mentioned groups of disorders, and in spite of the discrepancies mentioned above, we can anticipate that a PPI deficit can be considered a common process among multiple neuropsychiatric disorders. However, to the best of our knowledge, there are no studies on PPI using a transdiagnostic approach. Therefore, this review aims to systematically analyze the transdiagnostic status of PPI deficit in trauma-, stress-, and anxiety-related, mood-related, neurocognitive and other disorders such as obsessive-compulsive, tic-related, and substance use disorders.

## Method

### Search strategy and eligibility criteria

This systematic review was performed following the standards of the Preferred Reporting Items for Systematic Reviews and Meta-Analyses (PRISMA) statement [76] (PRISMA checklist is presented in Table S1 in the Online Resource). The protocol was registered in the International Prospective Register of Systematic Reviews (PROSPERO) on April 30th, 2022 (registration number: CRD42022322031). To identify relevant documents, an initial search was conducted on February 1st, 2022, in three bibliographic electronic databases: PubMed, PsycInfo, and Web of Science (WoS). This comprehensive search was updated on the last day of each month from February 1st (2022), to identify new studies published from inception to the present. The last search was conducted on May 31st, 2023.

The search strategy (see Table 1) included three main query fields: PPI; (AND) the target groups of neuropsychiatric disorders (trauma, stress and anxiety-related, mood-related, neurocognitive and other disorders, such as obsessive-compulsive, tic-related, and substance use disorders); and (NOT) animal models. Therefore, we searched for studies that evaluated PPI in human populations diagnosed with any of the target disorders.

**Table 1:** Search strategy

Query	Field	Search term
#1	Title/Abstract/ Keywords	“Prepulse inhibition” OR “Pre-pulse inhibition”
#2	Title/Abstract/ Keywords	“Posttraumatic stress disorder” OR PTSD OR Anxiety OR Stress OR “Panic disorder” OR Alzheimer OR Huntington OR Parkinson OR Depression OR “Major depressive disorder” OR “Bipolar disorder” OR “Obsessive compulsive disorder” OR OCD OR “Gilles de la Tourette syndrome” OR “Tourette syndrome” OR GTS OR “Substance-related disorders” OR Addiction OR “Drug use*” OR “Cannabis use*” OR “Cocaine use*” OR “MDMA use*” OR “Alcohol use*”
#3	Title	“Animal model” OR Mice OR Mouse OR Rat OR Rats OR Fish OR Fishes OR Rodent OR Rodents OR Monkey OR Monkeys
#4	N/A	(#1 AND #2) NOT #3

**Note**: Abbreviations: N/A, not applicable

The neuropsychiatric disorder groups included in the search strategy were chosen instead of others where a PPI deficit has been more or less consistently observed, such as the spectrum of schizophrenia disorders, autism spectrum disorders, and neurodevelopmental disorders. This is because recent meta-analyses and systematic reviews have been published on the deficit in sensorimotor gating in the schizophrenia spectrum (San-Martin et al., 2020 [58]), high-risk mental states (Li et al., 2020 [59]), autism spectrum (Cheng et al., 2018 [62]), and neurodevelopmental disorders (Schulz et al., 2023 [63]). Following the PRISMA guidelines, as well as the good practices associated with conducting reviews, the duplication of reviews on a topic for which there are already previous and recent reviews should be avoided (e.g., Higgins and Green, 2011 [77]; Petticrew, 2015 [78]). Therefore, these mentioned disorder groups, for which systematic and integrative searches already exist, were not included in this review.

A primary literature mapping was performed using the terms “prepulse inhibition” and “neuropsychiatric disorders” in PubMed and PsycInfo databases allowing the selection of inclusion and exclusion criteria (see Table S2 in the Online Resource for full criteria). To be included in the review, studies had to meet the following inclusion criteria: sample of any age diagnosed with or meeting the diagnostic criteria for one of the target disorders, as well as studies where participants were exposed to an experimental induction of these conditions; intervention consisted of an assessment of startle response and PPI using an experimental task; control groups included undiagnosed participants as well as persons not exposed to an experimental induction; and finally, the studies reported startle response and PPI data. Studies written in English or Spanish, meeting these criteria, and reporting experimental case-control designs were included.

As for exclusion criteria, studies were excluded if they were written in a language different from English or Spanish, did not assess PPI or startle response, participants did not correspond to any of the groups of disorders selected for the analysis, they were only focused on animal models, did not have a control group, or did not report an experimental case-control design.

### Study selection

The study selection was performed by independent peer review, with a third independent reviewer resolving disagreements. It was carried out in two stages: first, reading the title, abstract, and keywords of the identified records (inter-rater reliability was acceptable, with an 83.49% of agreement). Second, two independent reviewers read the selected records. Again, the inter-rater reliability was acceptable (74.72% agreement), with a third reviewer resolving the disagreements. Additionally, a snowballing approach was implemented to map eligible articles that had not appeared in the search engines. This process was performed at two levels: searching among the reference lists of systematic reviews and meta-analyses identified in the screening, and among the primary references of the records included after this process.

### Data extraction

A data extraction form was designed and can be found in Table S3 in the Online Resource. The main characteristics of the records were extracted through independent peer review. From each record, we obtained bibliographic data, group characteristics (mean age, sex, and total number of participants), objectives, experimental design, method to register the startle response and PPI, results, and conclusions. Mendeley (version 1.19.8), Parsifal (parsif.al), and Excel (version 16.43) were used to manage the references and records.

### Assessment of risk of bias

Study quality and risk of bias of the records were independently peer-reviewed using the Newcastle-Ottawa Scale (NOS) for case-control studies [79], with a third independent reviewer resolving disagreements. This scale assesses the categories of selection, comparability, and exposure, with star-shaped scores ranging from 0 to 9, with higher scores indicating a lower risk of bias [80]. Following the PRISMA guidelines [76] and the Cochrane Handbook for Systematic Reviews and Meta-analysis [77], studies will not be excluded from the systematic analysis of results unless they are scored with a very high risk of bias (e.g., a score of 0–3 stars on the NOS scale). This premise is followed because, as Petticrew reported (2015 [78]), even “weaker” studies in terms of evidence can provide valuable information for the context of a systematic and integrative review of the scientific literature.

## Results

### Study selection and characteristics

**Fig. 1:**
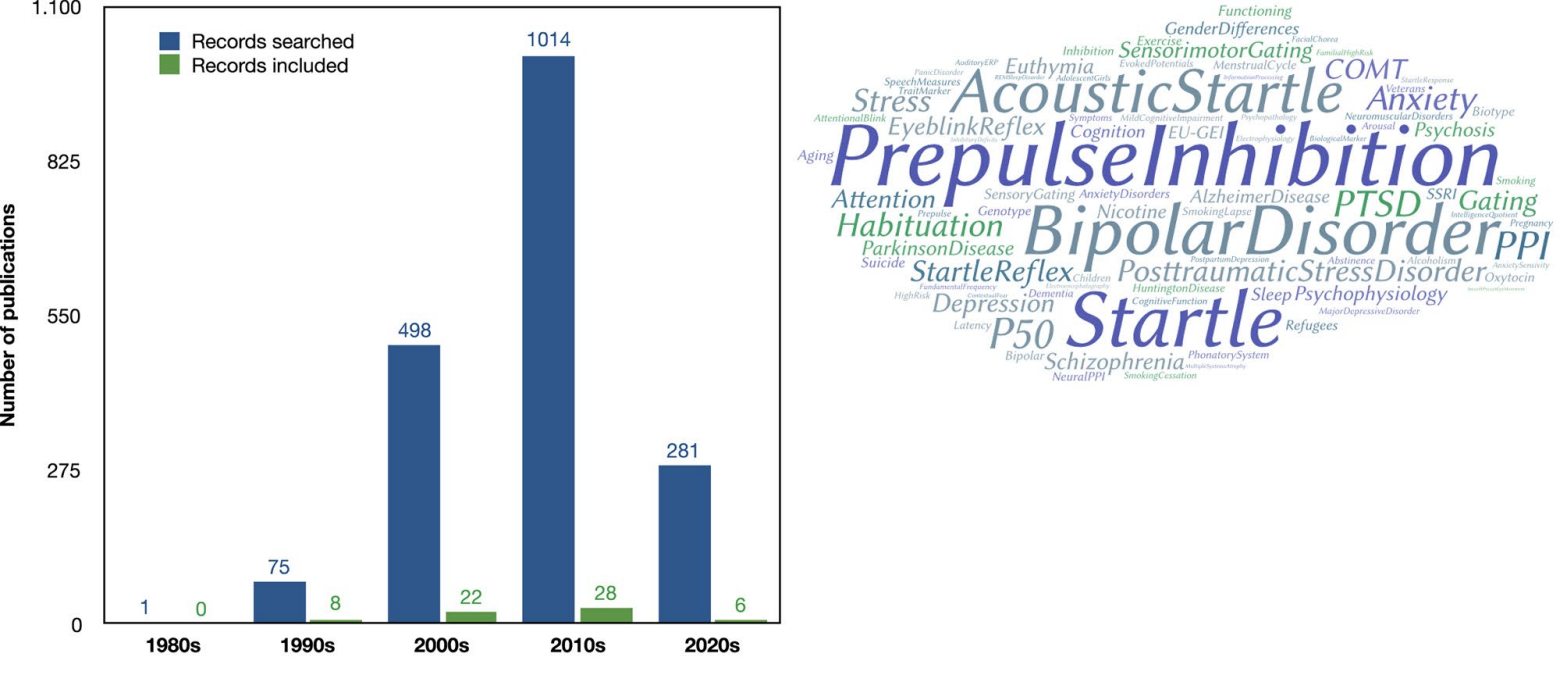
Evolution (left side) and word cloud of the most used keywords in the identified records (right side). Note: The time evolution graph is divided by sections in the decades: 1980–1989; 1990–1999; 2000–2009; 2010–2019. The 2020s decade corresponds to the years 2020-23

Among the records identified in the preliminary search, an upward trend has been observed in the number of records published from 1986 to the present. A decadal view (see Fig. 1 – left side) shows the temporal evolution of the number of publications. Figure 1 (right side) depicts the most frequently used keywords.

**Fig. 2:**
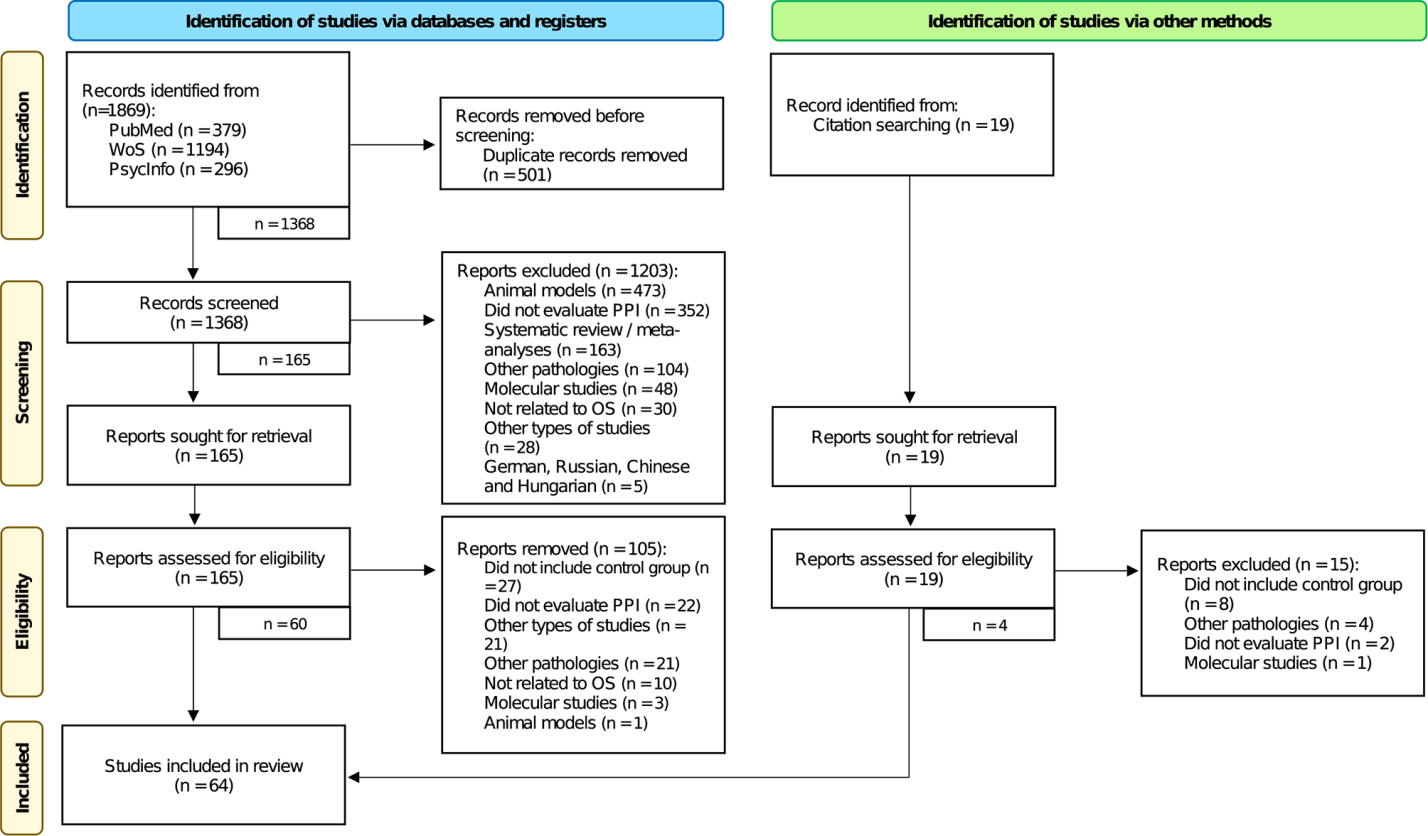
PRISMA flowchart. Notes: (1) PPI: prepulse inhibition; n: number of records; OS: object of study; OCD: obsessive-compulsive disorder; (2) From Page et al. [76]. Distributed under the terms of the Creative Commons Attribution License

The systematic search initially identified a total number of 1.869 records. After removing duplicates, 1.368 records were screened through the reading of titles, abstracts and keywords. A total of 1.203 studies were excluded for different reasons, mainly because they focused on animal models, did not evaluate PPI, or were systematic reviews and/or meta-analyses. Thus, 165 studies were selected for full-text retrieval and subsequent eligibility assessment. Of these, 60 records met the inclusion criteria. Four studies that were identified through the snowballing approach were also included, reaching 64 studies. Figure 2 shows a detailed flowchart of the process and the causes of exclusion.

### General data description

A summary of the main sociodemographic data extracted from the studies is presented in Table 2. The overall mean sample size was 109.72 participants (SD = 209.06), with a smaller sample size in the patient groups than in the controls (Mean = 36.69, SD = 47.1, and Mean = 56.03, SD = 151.19, respectively). The mean age of the patients was 35.28 years (SD = 14.04), and 34.14 (SD = 13.42) for the control groups. As for the gender, there were, on average, fewer women than men in both the control (43.73%, and 56.27%, respectively), and patient groups (44.42%, and 55.58%, respectively).

**Table 2:** Sociodemographic data and experimental conditions of included studies

Study	N total (% female)	Mean age (SD)	Diagnosis	ISI (ms)	Startle stimuli / Prepulse stimuli
Swerdlow et al. [84]	44 (32%)	45 (2.9)	HD	30, 60, 120	116dB (40ms) / 85dB (20ms)
Grillon et al. [85]	48 (0%)	41.3 (5)	PTSD	120	98-103dB (40ms) / 70dB (30ms)
Grillon et al. [86]	66 (53%)	13.6 (NR)	Anxious sensitivity	120, 4000	106dB (40ms) / 70dB (30ms)
Grillon et al. [81]	65 (0%)	44.2 (4)	PTSD	120	93-103dB (40ms) / 70dB (30ms)
Grillon et al. [82]	52 (0%)	48 (3.7)	PTSD	120	103dB (40ms) / 70dB (30ms)
Perry et al. [87]	48 (38%)	34.6 (11.7)	BD	30, 60, 120	115dB (40ms) / 86dB (20ms)
Ludewig et al. [88]	41 (49%)	35.9 (10)	PD	30, 60, 120, 240	115dB (40ms) / 86dB (20ms)
Muñoz et al. [89]	32 (NR)	47.4 (10.4)	HD	50, 70, 100, 150	A: 90dB / 80dB; T: 3xU / 1.5xU
Hejl et al. [90]	97 (59%)	72.6 (5.3)	AD, MCI	30, 60, 120	115dB (40ms) / 85dB (40ms)
Perry et al. [91]	46 (52%)	34 (9.3)	MDD	30, 60, 120	118dB (40ms) / 72-86dB (20ms)
Perriol et al. [92]	40 (NR)	73.3 (NR)	PD, AD	60, 120, 3000	115dB (40ms) / 80dB (40ms)
Rich et al. [93]	29 (55%)	12.9 (2.5)	BD	60, 120	104dB (50ms) / 70dB (50ms)
Barrett et al. [94]	43 (49%)	44.3 (13.2)	BD	60, 120	111dB (40ms) / 75-85dB (40ms)
Ludewig et al. [95]	42 (50%)	34.5 (10)	PD	30, 60, 120, 240	115dB (40ms) / 86dB (20ms)
Lipschitz et al. [96]	51 (100%)	16.5 (2.8)	PTSD	120, 2000	104dB (40ms) / 72dB (40ms)
Quednow et al. [97]	38 (42%)	35.2 (10.8)	MDD, Dysthymia	140	116dB (40ms) / 86dB (20ms)
Ueki et al. [98]	70 (69%)	70.2 (8.6)	AD, MCI	50	115dB (50ms) / 85dB (30ms)
Carroll et al. [99]	67 (51%)	34.5 (8.2)	BD	120	95dB (50ms) / 65dB (50ms)
Giakoumaki et al. [100]	57 (NR)	32 (7.2)	BD	60, 120	115dB (40ms) / 85dB (20ms)
Duley et al. [101]	38 (61%)	21.2 (0.4)	Trait anxiety	30, 60, 120	102dB (40ms) / 70dB (40ms)
Gogos et al. [102]	61 (51%)	41.7 (11.3)	BD	60, 120	115dB (40ms) / 74-86dB (20ms)
Holstein et al. [103]	51 (76%)	38.7 (2.2)	PTSD	60, 120, 2000	115dB (40ms) / 86dB (20ms)
McMillan et al. [104]	50 (76%)	22.9 (5.8)	Anxious sensitivity	120	105dB (50ms) / 70dB (25ms)
Vrana et al. [105]	100 (50%)	42 (10.8)	PTSD	60, 120, 240	100dB (50ms) / 70dB (20ms)
Zoetmulder et al. [106]	82 (49%)	59.7 (9.1)	PD	30, 60, 120, 300	115dB (40ms) / 75-85dB (20ms)
Ivleva et al. [107]	214 (56%)	34.3 (11.7)	BD	120, 4500	116dB (40ms) / 80dB (20ms)
Comasco et al. [108]	204 (100%)	30.4 (4.9)	Trait anxiety	100	115dB (40ms) / 72-86dB (20ms)
Vrana et al. [109]	95 (53%)	42 (10.8)	PTSD	60, 120, 240	100dB (50ms) / 70dB (20ms)
Sánchez-Morla et al. [110]	102 (56%)	40.9 (10.5)	BD	60, 120	118dB (40ms) / 80dB (20ms)
Pineles et al. [111]	47 (100%)	31.9 (9.2)	PTSD	120	100dB (50ms) / 70dB (20ms)
De la Casa et al. [83]	22 (64%)	21 (NR)	Stress induction	40, 60, 80	95dB (20ms) / 75dB (50ms)
Comasco et al. [112]	170 (100%)	30.9 (4.8)	Postpartum MDD	100	115dB (40ms) / 72-86dB (20ms)
Echiverri-Cohen et al. [113]	67 (67%)	32.7 (13.4)	PTSD	30, 60, 120	105dB (50ms) / 75dB (25ms)
Matsuo et al. [114]	471 (53%)	39.9 (11.7)	MDD	60, 120	115dB (40ms) / 86-90dB (20ms)
Millian-Morell et al. [115]	87 (48%)	70.3 (11.9)	PD	60, 120, 1000	115dB (40ms) / 85dB (20ms)
Meteran et al. [116]	45 (47%)	45.9 (13.1)	PTSD	60, 120	115dB (20ms) / 76-85dB (20ms)
Bo et al. [117]	63 (40%)	26.3 (6.7)	BD	120	100dB (40ms) / 65dB (150ms)
Matsuo et al. [118]	338 (59%)	40.4 (11.1)	BD	60, 120	115dB (NR) / 86-90dB (NR)
Massa et al. [119]	1143 (59%)	38.6 (14)	BD	120	116dB (40ms) / 90dB (20ms)
Storozheva et al. [120]	240 (47%)	33.2 (1.2)	GAD	60, 120, 2500	110dB (40ms) / 85dB (20ms)
San-Martin et al. [121]	44 (39%)	26.6 (7.7)	BD	30, 60, 120	115dB (40ms) / 85dB (20ms)
Acheson et al. [122]	1228 (0%)	22.2 (2.87)	PTSD	30, 60, 120	114dB (40ms) / 86dB (20ms)
Swerdlow et al. [123]	24 (46%)	36.3 (5.5)	OCD	100	116dB (40ms) / 72-86dB (20ms)
Castellanos et al. [124]	21 (0%)	10.8 (2)	GTS	30, 60, 90, 120, 250	9.0mA (NR) / 6.0mA (NR)
Swerdlow et al. [125]	24 (33%)	12 (NR)	GTS	120	105dB (40ms) / 86dB (20ms)
Hoenig et al. [126]	60 (50%)	31.5 (1.7)	OCD	120	116dB (40ms) / 72-86dB (20ms)
de Leeuw et al. [127]	50 (72%)	32.4 (9.7)	OCD	120	113dB (30ms) / 74-86dB (30ms)
Ahmari et al. [128]	44 (45%)	31 (9)	OCD	120	116dB (40ms) / 74-86dB (20ms)
Buse et al. [129]	44 (0%)	13.7 (1.8)	GTS	140	40psi (40ms) / 6psi (20ms)
Kohl et al. [130]	24 (44%)	39.7 (11.6)	OCD	60, 120, 200	110dB (20ms) / 80dB (20ms)
Zebardast et al. [131]	33 (52%)	30.4 (9.3)	GTS	120	80psi (40ms) / 7psi (20ms)
Pittenger et al. [132]	24 (66,7%)	30.9 (2.5)	OCD	120	102dB (50ms) / 85dB (5ms)
Steinman et al. [133]	110 (50%)	28.2 (6.2)	OCD	120	116dB (40ms) / 74-86dB (20ms)
Efferen et al. [134]	24 (0%)	42.4 (2.9)	CUD	100	115dB (40ms) / 75-85dB (20ms)
Quednow et al. [135]	50 (0%)	24.4 (5.1)	EUD, CnUD	120	116dB (40ms) / 72-86dB (40ms)
Heekeren et al. [136]	43 (21%)	26.3 (3.6)	EUD	100	115dB (20ms) / 82dB (20ms)
Kedzior et al. [137]	28 (20%)	31.5 (8)	CnUD	20, 40, 80, 100, 200	100dB (50ms) / 70dB (20ms)
Kedzior et al. [138]	36 (17%)	32.2 (7.5)	CnUD	20, 40, 80, 100, 200	100dB (50ms) / 70dB (20ms)
Mathias et al. [139]	78 (24%)	16.1 (0.9)	CnUD	120	105dB (50ms) / 70-85dB (50ms)
Preller et al. [140]	159 (27%)	31.6 (9.1)	CUD	30, 60, 120, 240	115dB (40ms) / 86dB (20ms)
Winton-Brown et al. [141]	47 (55%)	22.7 (3.7)	CnUD	30, 60, 120, 1000	114dB (40ms) / 85dB (20ms)
Morales-Muñoz et al. [142]	43 (37%)	26.5 (2.6)	CnUD	30, 60, 120	100dB (40ms) / 30dB (30ms)
Gil-Miravet et al. [143]	74 (0%)	42.3 (8.5)	CUD	30, 60, 120	105dB (40ms) / 85dB (20ms)
Echevarria et al. [144]	44 (0%)	40.7 (10)	CUD	30, 60, 120	105dB (40ms) / 85dB (20ms)

**Note**: Abbreviations: AD, Alzheimer’s disease; BD, bipolar disorder; dB, decibels; CnUD, cannabis-use disorder; CUD, cocaine-use disorder; EUD, ecstasy-use disorder; GAD, general anxiety disorder; GTS, Gilles de la Tourette syndrome; HD, Huntington’s disease; ISI, interstimulus interval; mA, milliamperes; MCI, mild cognitive impairment; MDD, major depressive disorder; ms, milliseconds; N, number of participants; NR, not reported; OCD, obsessive compulsive disorder; PD, panic disorder; PD, Parkinson’s disease; psi, pounds per square inch; PTSD, post-traumatic stress disorder; SD, standard deviation

The psychopathological scales more used were the structured clinical interviews of the DSM-III (9.4%) and DSM-IV (40.6%), and the diagnostic criteria of the International Classification of Diseases (10.9%). Other psychometric scales used were the Yale-Brown Obsessive Compulsive Scale (12.5%), the Clinician-Administered PTSD Scale (7.8%), the Yale Global Tic Severity Scale (4.7%), and the State-Trait Anxiety Test (3.1%). In half of the studies, it was reported the use of psychopharmacology in the pathological sample (53.1%, 34/64), being antidepressants (20.3%) and anxiolytics (6.2%) the most common.

Nineteen studies included patients diagnosed with trauma-, stress- and anxiety-related disorders (29.7%), sixteen included mood disorders (25%), seven evaluated patients with neurocognitive disorders (10.9%), and twenty-two included other disorders (obsessive-compulsive, tic-related and substance-use disorders) (34.4%). All studies had a case-control design evaluating startle response and PPI, including participants with some psychopathology (98.44%), while the control groups were composed of healthy subjects. In three studies [81–83], participants were exposed to an experimental induction of emotions, but the control group did not receive such induction.

### Experimental conditions

The experimental conditions are summarized in Table 2. Most of the studies used more than one interstimulus interval between the prepulse and pulse (60.9%, 39/64), with 120 ms (81.2%), 60 ms (51.6%), and 30 ms (26.6%) being the most common intervals. The overall mean pulse intensity was 109.59 dB (SD = 7.23; range 90–118), with a mean duration of 40.5 ms (SD = 6.93; range 20–50). The mean prepulse duration was 27.1 ms (SD = 18.61; range 5-150), with a mean intensity of 78.54 dB (SD = 6.91; range 65–90). All experiments registered the electromyographic response of the orbicularis oculi muscle as the measure of the startle response. Three of the studies recorded it bilaterally [101, 120, 125], while the remaining studies recorded it on the right (57.8%, 37/64) or left orbicularis muscle (23.4%, 15/64). All studies used an acoustic sensory modality, with five studies using also tactile stimuli [84, 89, 124, 129, 131].

### Trauma-, stress-, and anxiety-related disorders

The summary of the main findings from the studies on trauma-, stress-, and anxiety-related disorders appears in Table 3. The studies included PTSD (n = 11, 57.9%), panic disorder (n = 2, 10.5%), trait anxiety (n = 2, 10.5%), anxious vulnerability (n = 2, 10.5%), and generalized anxiety disorder (n = 1, 5.3%) patients. One study exposed subjects to an experimental induction of stress (5.3%).

**Table 3:** Summary of studies on PPI deficit that compared trauma-, stress-, and anxiety-related disorders patients to matched controls

Study	Startle response (SR)	Prepulse inhibition (PPI)	Neurobiology proposed	Cognition proposed
Storozheva et al. [120]	GAD > Control: ↓ SR mg. ↑ SR lat.	GAD > Control: ↓ %PPI (ISI 60ms)	GAD > Control: ↑ LH RT ↓ SR ↓ PFC RT ↓ PPI	Misinterpretation of contextual cues RT ↓ PPI
Grillon et al. [85]	No differences	PTSD > Control: ↓ %PPI	NR	Affective flattening, avoidance & re-experiencing RT ↓ PPI
Grillon et al. [81]	S1: No differences S2: PTSD > Control: ↑ SR	PTSD > Control: ↓ %PPI	↑ Activity of the NST & hippocampus RT ↓ PPI	Re-experiencing RT ↑ SR mg.
Grillon et al. [82]	PTSD > Control: ↑ SR mg.	No differences	↑ Activity of the NST & hippocampus RT ↑ SR mg.	Re-experiencing & avoidance RT ↑ SR mg.
Lipschitz et al. [96]	No differences	No differences	NR	NR
Holstein et al. [103]	PTSD > Control: ↑ SR mg.	No differences	NR	NR
Vrana et al. [105]	PTSD > Control: ↓ SR lat. ↑ SR mg.	PTSD > Control: ↓ %PPI (ISI 60 and 120ms)	NR	Hypervigilance RT SR ↓ lat. & ↑ mg.
Vrana et al. [109]	PTSD > Control: ↑ SR mg.	PTSD > Control: ↓ %PPI	PFC deficit RT ↓ PPI	↑ Abstinence & planning RT ↑ PPI
Pineles et al. [111]	PTSD > Control: ↓ SR mg.	PTSD > Control: ↓ %PPI	NR	Re-experiencing & avoidance RT ↓ PPI
Echiverri-Cohen et al. [113]	No differences	PTSD > Control: ↓ %PPI (ISI 30 and 60ms)	NR	Re-experiencing & avoidance RT ↓ PPI
Meteran et al. [116]	PTSD > Control: ↑ SR mg.	No differences	PFC deficit RT ↑ SR	Hallucinations & other psychotic symptoms RT ↑ SR
Acheson et al. [122]	No differences	PTSD > Control: ↓ %PPI (ISI 30 and 60ms)	PFC, hippocampus & amygdala deficit RT ↓ PPI	Stress RT ↓ PPI
De la Casa et al. [83]	Stress > Control: ↓ SR mg.	Stress > Control: ↓ %PPI (ISI 60 and 80ms)	↑ Dopaminergic activity RT stress induction RT ↓ PPI	NR
Duley et al. [101]	NR	TA > Control: ↓ %PPI	NR	Exercise modulates PPI deficit in anxiety
Comasco et al. [108]	PW > Control: ↑ SR mg.	PW > Control: ↓ %PPI PW + TA > PW: ↓ %PPI PW + TA + SSRI > PW + TA: ↓ %PPI	↑ Estrogens & catecholaminergic genotype RT ↓ PPI	Attention & executive deficits RT ↓ PPI
Grillon et al. [86]	AS > Control: ↑ SR mg.	AS > Control: ↓ %PPI	NR	NR
McMillan et al. [104]	AS > Control: ↑ SR mg.	AS > Control: ↓ %PPI	NR	Difficulty disengaging attention RT ↓ PPI
Ludewig et al. [88]	No differences	PD > Control: ↓ %PPI PD + A > PD-A: ↓ %PPI	NR	Trait anxiety RT ↓ PPI
Ludewig et al. [95]	PDnM > Control: ↑ SR mg.	PDnM > Control: ↓ %PPI PDnM > PDM: ↓ %PPI (240ms)	↑ Dopaminergic & amygdala activity RT ↓ PPI	Deficit in interpreting somatic symptoms RT ↑ SR

**Note**: Abbreviations: ↑, increase or hyperactivation of; ↓, deficit or hypoactivation of; AS, anxious sensitivity; GAD, general anxiety disorder; ISI, interstimulus interval; Lat., latency; Mg., magnitude; ms, milliseconds; NR, not reported; NST, nucleus of the stria terminalis; PD, panic disorder; PD + A/-A, panic disorders patients with or without anxiety; PDM/nM, medicated or unmedicated panic disorder patients; PFC, prefrontal cortex; PTSD, post-traumatic stress disorder; PW, pregnant women; PW + TA, pregnant women with trait anxiety; PW + TA + SSRI, pregnant women with trait anxiety medicated with selective serotonin reuptake inhibitors; RH, left hemisphere; RT, related to; S1/2, first and second sessions; PPI, prepulse inhibition; SR, startle response; TA, trait anxiety

Regarding startle response intensity, most studies found a higher magnitude of startle response for the patients than the control group (52.6%), whereas fewer studies reported a lower magnitude (15.8%). Five studies did not find differences between groups [85, 88, 96, 113, 122] and one study did not report data on startle magnitude [101]. More specifically, an increased startle response appeared for PTSD [81, 82, 103, 105, 109, 116] and for anxious vulnerability patients [86, 104].

PPI results were more consistent, with 15 of the 19 studies in this group reporting disrupted PPI in the pathological group compared to the control group (78.9%). The studies without differences in PPI only included patients with PTSD [82, 96, 103, 116].

### Mood disorders

A detailed analysis of all the variables for each study included in this group of disorders is presented in Table 4. The studies included bipolar disorder (n = 12, 75%), major depressive disorder (n = 4, 25%), and dysthymia (n = 1, 6.25%).

**Table 4:** Summary of studies on PPI deficit that compared mood-related disorders patients to matched controls

Study	Startle response (SR)	Prepulse inhibition (PPI)	Neurobiology proposed	Cognition proposed
Perry et al. [91]	No differences	No differences (tendency: MDD > Control: ↓%PPI)	CSPP deficit RT ↓ PPI	NR
Quednow et al. [97]	No differences	No differences	NR	Suicide attempt no RT PPI
Comasco et al. [112]	MDD > Control: ↑ SR mg.	MDD > Control: ↓ %PPI	Genetic risk RT ↓ PPI	Depression & insomnia RT ↓ PPI
Matsuo et al. [114]	No differences	MDD♂>Control♂: ↓ %PPI	Sexual dimorphism RT PPI	Depression RT ↓ PPI
Perry et al. [87]	No differences	BD > Control: ↓ %PPI	CSPP deficit RT ↓ PPI	Cognitive fragmentation RT ↓ PPI
Rich et al. [93]	No differences	No differences	NR	ADHD symptoms no RT PPI
Barrett et al. [94]	No differences	No differences	NR	NR
Carroll et al. [99]	BD > Control: SR ↓mg. ↑lat.	No differences	NR	Depression RT ↑ SR
Giakoumaki et al. [100]	No differences	BD > grBD > Control: ↓ %PPI	Genetic risk & PFC deficit RT ↓ PPI	↓ Inhibitory control RT ↓ PPI
Gogos et al. [102]	BD♂>Control♂: ↓ SR mg.	BD♂>Control♂: ↓%PPI (60ms) BD♀>Control♀: ↑%PPI (120ms)	Increase in 5-HT receptors RT ↑ PPI (♀)	↑ ISI (120ms) mobilizes attentional resources
Ivleva et al. [107]	No differences	No differences	NR	NR
Sánchez-Morla et al. [110]	BD > Control: ↑ SR lat.	BD > Control: ↓ %PPI (ISI 60 and 120ms)	Amygdala déficit RT ↓ PPI	↓ Social cognition RT ↓ PPI
Bo et al. [117]	No differences	BD > Control: ↓ %PPI	PFC deficit RT ↓ PPI	↓ Inhibitory control RT ↓ PPI
Matsuo et al. [118]	No differences	BD♂>Control♂: ↓ %PPI	NR	Depression RT ↓ PPI
Massa et al. [119]	BD > Control: ↑ SR lat.	No differences	Genetic risk RT ↓ PPI	↓ Memory, executive function & SIP RT ↓ SR
San-Martin et al. [121]	No differences	BD > Control: ↓%PPI (ISI 60ms)	NR	NR

**Note**: Abbreviations: ↑, increase or hyperactivation of; ↓, deficit or hypoactivation of; BD, bipolar disorder; CSPP, corticostriatal-pallidopontine limbic circuit; ISI, interstimulus interval; Lat., latency; MDD, major depressive disorder; Mg., magnitude; ms, milliseconds; NR, not reported; PFC, prefrontal cortex; PPI, prepulse inhibition; RT, related to; SIP, speed of information processing; SR, startle response

Regarding startle response intensity, most studies did not find differences between the groups (n = 11, 68.75%), two studies found a reduced magnitude between patients as compared with controls [99, 102], and another one reported an increased magnitude for bipolar disorder patients [96]. By other hand, three studies reported longer latencies of the startle response in the groups of patients [99, 110, 119].

Nine studies (56.25%) revealed reduced PPI in patients compared to the control group. Specifically, 7 of 12 studies with bipolar disorder patients informed of a PPI deficit [87, 100, 102, 110, 117, 118, 121]. Of the remaining studies, four records did not find any differences between the groups [93, 94, 99, 119], and one study reported reduced PPI in women from the control group compared to bipolar patients [102].

The results from studies with major depressive disorder patients were quite contradictory. Thus, two of the studies found disrupted PPI for major depressive disorder patients when compared with control group participants [112, 114], and another two studies did not find differences between groups [91, 97].

### Neurocognitive disorders

This group of studies included patients with Parkinson’s (n = 3, 42.8%), Alzheimer’s (n = 3, 42.8%) and Huntington’s (n = 2, 28.6%) diseases. Two additional studies included patients with mild cognitive impairment. A detailed analysis of all the variables for each study included in this group of disorders is depicted in Table 5.

**Table 5:** Summary of studies on PPI deficit that compared neurocognitive disorders patients to matched controls

Study	Startle response (SR)	Prepulse inhibition (PPI)	Neurobiology proposed	Cognition proposed
Perriol et al. [92]	NR	PD > AD > Control: ↓ %PPI (ISI 120ms)	Subcortical-thalamo-cortical system dysfunction RT ↓ PPI	Exogenous care RT PPI modulation (ISI 120ms)
Zoetmulder et al. [106]	No differences	PD > Control: ↓ %PPI (ISI 60 and 120ms)	Striatal dysfunction RT ↓ PPI	NR
Millian-Morell et al. [115]	No differences (tendency: PD > Control: ↑ SR lat.)	PD > Control: ↑ %PPI (ISI 120ms)	Deficits in basal ganglia, PFC & dopaminergic network RT ↑ PPI	Motor coordination deficit RT ↑ PPI
Hejl et al. [90]	No differences	No differences	The cholinergic system would have a weak relationship with PPI	NR
Ueki et al. [98]	No differences	MCI > Control: ↑ %PPI AD > Control: ↓ %PPI	Deficits in entorhinal cortex in early stages of AD RT ↓ PPI	Cognitive-behavioral dementia symptoms RT ↓ PPI
Swerdlow et al. [84]	HD > Control: ↑ SR lat.	HD > Control: ↓ %PPI	Deficits in GABA efferent circuit from striatum-pale RT ↓ PPI	Inhibitory & executive deficits RT ↑ SR latency
Muñoz et al. [89]	HD > Control: ↑ SR lat.	HD > Control: ↓ %PPI HD + cm > HD: ↓ %PPI	Glutamatergic dysfunction RT ↓ PPI	NR

**Note**: Abbreviations: ↑, increasement or hyperactivation of; ↓, deficit or hypoactivation of; BD, bipolar disorder; AD, Alzheimer’s disease; HD, Huntington’s disease; HD + cm: Huntinton’s disease patients with choreic movements; ISI, interstimulus interval; Lat., latency; MCI, mild cognitive impairment; ms, milliseconds; NR, not reported; PD, Parkinson’s disease; PFC, prefrontal cortex; PPI, prepulse inhibition; RT, related to; SR, startle response

Regarding startle response, 3 studies (42.8%) reported longer latencies for the patients, and 3 (42.8%) did not find differences between groups. As for PPI results, 71.4% of the studies reported reduced PPI in the group of patients compared to the control group, and only one study did not find any differences [90]. Studies including patients with movement disorders showed the most consistent results. Specifically, all studies including patients with Huntington’s disease found reduced PPI [84, 89], with a greater deficit in patients with chorea [89]. Consistency was also high for Parkinson’s disease patients, showing a generalized PPI deficit [92, 106], except for one study by Millian-Morell et al. [115] which reported an increase in PPI.

Regarding Alzheimer’s type dementia, two studies found reduced PPI [92, 98], and one study did not find significant differences [90]. Patients with mild cognitive impairment either did not differ from [90] or showed increased PPI compared to controls [98].

#### Other Disorders

Obsessive-compulsive, tic-related and substance-use disorders.

In this group of disorders, patients with obsessive-compulsive disorder (n = 7, 31.8%), Gilles de la Tourette syndrome (n = 4, 18.2%), and substance use disorders (n = 11, 50%) were included. Specifically, the included addictive disorders referred to the use of cannabis (n = 5), cocaine (n = 4), and ecstasy (n = 2). A comprehensive analysis of all variables analyzed in relation to these studies can be seen in Table 6. Next, an analysis of the startle response and prepulse inhibition will be conducted separately for each group.

**Table 6:** Summary of studies on PPI deficit that compared obsessive-compulsive, Tourette and substance-use disorders patients to matched controls

Study	Startle response (SR)	Prepulse inhibition (PPI)	Neurobiology proposed	Cognition proposed
Swerdlow et al. [123]	No differences	OCD > Control: ↓ %PPI	CSPP deficit RT ↓ PPI	Obsessive & compulsive (O-C) symptoms RT ↓ PPI
Hoenig et al. [126]	No differences	OCD > Control: ↓ %PPI	CSPP deficit RT ↓ PPI	O-C symptoms RT ↓ PPI
de Leeuw et al. [127]	NR	No differences	NR	NR
Ahmari et al. [128]	No differences	OCD > Control: ↓ %PPI	CSPP abnormalities RT ↓ PPI	O-C symptoms RT ↓ PPI History of tics RT ↓ PPI
Kohl et al. [130]	NR	OCD > Control: ↓ %PPI (ISI 60, 120 and 200ms)	NAcc deficit RT ↓ PPI	O-C symptoms’ severity RT ↓ PPI
Pittenger et al. [132]	NR	No differences	5HT1b receptor availability in basal ganglia & thalamus RT ↑ PPI	NR
Steinman et al. [133]	No differences	OCD♀>Control♀: ↓%PPI	NR	NR
Castellanos et al. [124]	No differences	GTS > Control: ↓ %PPI	Pallidal structures deficit RT ↓ PPI	Sensory experiences RT ↓ PPI
Swerdlow et al. [125]	No differences	GTS > Control: ↓ %PPI	NR	NR
Buse et al. [129]	No differences	GTS > Control: ↓ %PPI	Deficit in middle & postcentral gyrus, precuneus, cingulate cortex & caudate nucleus RT ↓ PPI	Tic severity RT ↓ PPI
Zebardast et al. [131]	No differences	No differences	Deficit in caudate nucleus, frontal cortex, anterior insula, cingulate cortex & middle gyrus RT ↓ PPI	NR
Efferen et al. [134]	CUD > Control: ↓ SR mg.	CUD > Control: ↑ %PPI (Prepulse intensity 75dB)	↓ Dopaminergic activity RT ↓ SR mg. & ↑ PPI	NR
Preller et al. [140]	No differences	CUD > Control: ↑ %PPI (ISI 120ms)	Alterations in catecholamine neurotransmission RT ↑ PPI	NR
Gil-Miravet et al. [143]	No differences	CUD > Control: ↑ %PPI (ISI 30ms)	D1 & D2 receptor expression RT differences in PPI	NR
Echevarria et al. [144]	NR	CUD > Control: ↑ %PPI (ISI 30ms)	NR	Psychopathic traits RT ↑ PPI
Heekeren et al. [136]	No differences	No differences	NR	NR
Quednow et al. [135]	No differences	EUD > Control: ↑ %PPI	Sensitivity/density of 5-HT2 and/or 5-HT1 RT ↑ PPI	NR
Kedzior et al. [137]	No differences	CnUD > Control: ↓ %PPI	NR	Attentional dysfunction RT ↓ PPI
Kedzior et al. [138]	No differences	CnUD > Control: ↓ %PPI	NR	Attentional dysfunction RT ↓ PPI
Mathias et al. [139]	No differences	CnUD > Control: ↓ %PPI	RT ↓ PPI	Deficits in sustained attention RT ↓ PPI
Winton-Brown et al. [141]	No differences	CnUD > Control: ↓ %PPI	NR	NR
Morales-Muñoz et al. [142]	NR	CnUD > Control: ↓ %PPI (ISI 30ms)	↑ Dopaminergic activity RT ↓ PPI	NR

**Note**: Abbreviations: ↑, increasement or hyperactivation of; ↓, deficit or hypoactivation of; CSPP, corticostriatal-pallidopontine limbic circuit; GTS, Gilles de la Tourette syndrome; ISI, interstimulus interval; Lat., latency; ms, milliseconds; NAcc, nucleus accumbens; NR, not reported; OCD, obsessive-compulsive disorder; PD, Parkinson’s disease; PFC, prefrontal cortex; PPI, prepulse inhibition; RT, related to; SR, startle response

Regarding obsessive-compulsive disorder, the majority of the reviewed studies did not find differences between the groups in terms of the startle response (57.1%), with some of them not reporting results (42.9%). Regarding PPI, 71.4% of the reviewed studies found that patients with obsessive-compulsive disorder exhibited a PPI deficit compared to controls [123, 126, 128, 130, 133], while two other studies did not find differences [127, 132].

In Gilles de la Tourette syndrome, no study found differences between groups when analyzing the startle response, although the majority reported a PPI deficit among patients (75%) [124, 125, 129], with a single study not finding differences in this measure [131].

With regard to the results from studies with substance use disorders patients, almost none of the studies found differences between patients and controls in the startle response (72.2%), except for one study that found a lower magnitude among patients using cocaine [134], and two studies that did not report data on this measure [142, 144]. Regarding PPI in patients using cannabis, all reviewed studies observed lower prepulse inhibition than subjects in the control group [137–139, 141, 142]. Conversely, in studies evaluating patients using cocaine, a generally higher PPI was found among patients compared to controls [134, 140, 143, 144]. Similarly, one of the reviewed studies analyzing ecstasy found the same trend [135] while another did not find any differences [136].

### Assessment of risk of bias

The results of the methodological quality analysis performed using the Newcastle-Ottawa Scale to assess the risk of bias of the studies included in the review are summarized in Table 7. In general, most of the studies had a low risk of methodological bias. The overall mean quality was 7.12 stars (SD = 0.97; range 5–9). Specifically, in the category of study selection, most of them presented a good definition of the cases, as well as representative samples of the population. The comparability analysis showed that most of the studies controlled for sex and age. Finally, the results of the level of exposure of the participants to the evaluation methods and the experimental paradigm revealed a moderate risk of bias, mainly due to the fact that many studies did not specify the non-response rate or the method of ascertainment for cases and controls. As none of the studies included in the review obtained a very high risk of bias score (NOS score of 0–3 stars), none of them were excluded from the qualitative analysis of the review.

**Table 7:** Risk of bias of the studies included in the systematic review assessed by Newcastle-Ottawa Quality Assessment Scale (NOS)

Study	Selection				Comparability	Exposure			Risk of bias assessment (0–9)
	Case definition	Represen-tativeness	Selection of controls	Definition of controls	Confounding factors (*)	Ascertainment of exposure	Same method of ascertainment	Nonresponse rate	
Swerdlow et al. [84]	*	*	*	*	*/*	*	*		8
Grillon et al. [85]	*	*	*	*	*/*	*	*		8
Grillon et al. [86]			*	*	*/*	*	*		6
Grillon et al. [81]	*		*	*	*/*	*	*	*	8
Grillon et al. [82]	*		*	*	*/*	*	*		7
Perry et al. [87]	*		*		*/*	*	*		6
Ludewig et al. [88]	*		*	*	*/*	*	*		7
Muñoz et al. [89]	*		*	*	*/	*	*		6
Hejl et al. [90]	*		*	*	*/*		*	*	7
Perry et al. [91]	*		*	*	*/*		*		6
Perriol et al. [92]	*		*		*/*	*	*		6
Rich et al. [93]	*		*	*	*/*	*	*		7
Barrett et al. [94]	*			*	*/*	*	*		6
Ludewig et al. [95]	*	*	*	*	*/*	*	*		8
Lipschitz et al. [96]	*		*		*/*	*	*		6
Quednow et al. [97]	*		*	*	*/*		*	*	7
Ueki et al. [98]	*	*	*	*	*/*	*	*		8
Carroll et al. [99]	*		*	*	*/*		*	*	7
Giakoumaki et al. [100]	*		*	*	*/*	*	*	*	8
Duley et al. [101]	*			*	*/*	*	*		6
Gogos et al. [102]	*		*	*	*/*	*	*		7
Holstein et al. [103]	*		*	*	*/*	*	*	*	8
McMillan et al. [104]	*		*	*	*/*		*		6
Vrana et al. [105]	*		*	*	*/*	*	*		7
Zoetmulder et al. [106]	*		*	*	*/*		*	*	7
Ivleva et al. [107]	*	*	*	*	*/*		*	*	8
Comasco et al. [108]	*	*	*	*	*/*	*	*	*	9
Vrana et al. [109]	*		*	*	*/	*	*		6
Sánchez-Morla et al. [110]	*		*	*	*/*		*		6
Pineles et al. [111]	*		*	*	*/*		*	*	7
De la Casa et al. [83]	*		*	*	*/*		*	*	7
Comasco et al. [112]	*	*	*	*	*/*	*	*		8
Echiverri-Cohen et al. [113]	*		*	*	*/*	*	*		7
Matsuo et al. [114]	*	*	*	*	*/*	*	*	*	9
Millian-Morell et al. [115]	*		*	*	*/*		*	*	7
Meteran et al. [116]	*		*	*	*/*	*	*	*	8
Bo et al. [117]	*		*	*	*/*	*	*		7
Matsuo et al. [118]	*	*	*	*	*/*	*	*	*	9
Massa et al. [119]	*	*	*	*	*/*	*	*	*	9
Storozheva et al. [120]	*	*		*	*/*	*	*	*	8
San-Martin et al. [121]	*		*	*	*/*	*	*		7
Acheson et al. [122]	*	*	*		*/*	*	*	*	8
Swerdlow et al. [123]	*		*	*	*/*	*	*		7
Castellanos et al. [124]	*		*	*	*/	*	*		6
Swerdlow et al. [125]	*		*		*/*	*	*	*	7
Hoenig et al. [126]	*		*	*	*/*	*	*		7
de Leeuw et al. [127]	*		*	*	*/*	*	*	*	8
Ahmari et al. [128]	*		*	*	*/*	*	*		7
Buse et al. [129]	*		*	*	*/*	*	*		7
Kohl et al. [130]	*		*	*	*/*		*		6
Zebardast et al. [131]	*		*		*/*	*	*		6
Pittenger et al. [132]	*		*	*	*/		*		5
Steinman et al. [133]	*	*	*	*	*/*	*	*	*	9
Efferen et al. [134]	*		*	*	*/	*	*		6
Quednow et al. [135]	*		*	*	*/*		*	*	7
Heekeren et al. [136]	*		*	*	*/	*	*		6
Kedzior et al. [137]	*		*	*	*/*	*	*		7
Kedzior et al. [138]	*		*	*	*/*	*	*		7
Mathias et al. [139]	*	*	*	*	*/*		*	*	8
Preller et al. [140]	*	*	*	*	*/*	*	*	*	9
Winton-Brown et al. [141]	*		*	*	*/*		*		6
Morales-Muñoz et al. [142]	*		*	*	*/*	*	*		7
Gil-Miravet et al. [143]	*		*	*	*/*	*	*	*	8
Echevarria et al. [144]	*		*	*	*/*	*	*		7

**Notes:** 1. * means a point in the category in which it is indicated; (2) Principal confounding factors were sex, age and smoking status

## Discussion

In this systematic review, we have reviewed and summarized all the available scientific evidence on PPI evaluation in neuropsychiatric disorders from a transdiagnostic perspective. More specifically, our main objective was to verify the potential role of PPI deficit as a transdiagnostic process in four groups of pathologies: (a) trauma-, stress- and anxiety-related; (b) mood-related; (c) neurocognitive; and d) other disorders. Considering the heterogeneity found in the revision between the different neuropsychiatric disorders, we will independently discuss the results for each group.

Regarding trauma, stress, and anxiety-related disorders, an increased startle response was reported in the group of patients for half of the studies reviewed (52.6%). On the other hand, the PPI deficit appeared in the patients group compared to their respective controls, except for some studies with PTSD patients (4/11). Specifically, reduced PPI appeared for panic disorder [88, 95], generalized anxiety disorder [120], anxious vulnerability [86, 104], trait anxiety [101, 108], and in non-pathological samples submitted to stress induction [83]. This high consistency is congruent with the fact that some factors that have been associated with a decrease in PPI, such as insomnia [145], or affective factors [51], plays a central role in these disorders [146].

In the mood disorders group, although no differences were found in the magnitude of the startle response in 68% of the studies, a reduction in PPI was reported for half of the reviewed reports. More specifically, in the case of bipolar disorder studies, a reduction in PPI has been obtained in nine of twelve studies. These data can be linked to some core factors in bipolar disorders, such as the presence of negative emotions or demotivation [147], which have also been related to a PPI reduction [148]. However, four studies didn’t find any differences between groups [93, 94, 99, 119], and another study reported reduced PPI in control women when compared with women diagnosed with bipolar disorder [102].

Regarding the studies that evaluated subjects with major depressive disorder, there was less consistency, since two studies reported a reduced PPI for major depressive disorder patients [96, 98] while another two studies did not report differences in PPI magnitude for patients with depression but without psychotic symptomatology [91, 97]. Similarly, those studies that reported reduced PPI included bipolar patients with psychotic symptoms [87, 121]. These findings support the idea that PPI could be modulated by active symptomatology [70].

Concerning the group of neurocognitive disorders, a general reduction in PPI magnitude was reported. Specifically, PPI was reduced for patients with Huntington’s, Parkinson’s, and Alzheimer’s diseases, with less consistency in the data corresponding to mild cognitive impairment patients. A possible explanation for such differences comes from the fact that PPI is gradually disrupted as the disease progresses, as is the case with other markers [149]. Therefore, PPI disruption can play a possible role as a biological marker in the differential diagnosis between the early and later stages of the disease [70]. Given that in this group of disorders it appears a progressive neurodegeneration of those areas controlling PPI, such as the hippocampus or medial prefrontal cortex [150, 151], the results carry weight.

Regarding the other reviewed disorders, there were no differences between patients and controls in startle response. However, differences were observed in terms of PPI. Thus, in obsessive-compulsive disorder (OCD), a deficit in PPI was reported in the majority of the reviewed studies (71.4%), both in medicated patients [123, 126] and unmedicated patients [128]. Furthermore, it was found that a greater deficit in PPI correlated with a history of tics [128] and with a higher severity of obsessive-compulsive symptoms [123, 126, 128, 130]. These findings are consistent with the results found in studies evaluating Gilles de la Tourette syndrome, where a widespread deficit in PPI is also present [124, 125, 129]. Considering that both disorders are related at the symptomatic level, it makes sense the hypothesis that the deficit in sensorimotor gating is functionally connected to the inability to inhibit repetitive thoughts and behaviors [68, 133].

Further inconsistencies are found in the group of substance use disorders. In the case of cannabis, a deficit in PPI is present in patients in almost all of the reviewed studies when compared to the control group, with the exception of one study in which patients had abstained from cannabis use for at least three days prior to PPI assessment [135]. This is consistent with some studies that have found experimentally decreased PPI with the administration of cannabinoid receptor agonists [152–154]. In other conditions, such as stimulant substance users, paradoxical effects have been observed among patients, who show elevated PPI levels despite cognitive deficits [140]. Regarding studies involving ecstasy users, one study found higher PPI among patients [135], while another study found no differences [136]. It has been hypothesized that this increase in PPI associated with stimulant drug use may be linked to changes in 5-HT receptors [135].

Regarding cocaine use, all the reviewed studies found higher PPI among patients compared to the control group. The fact that cocaine users have an increased PPI index may be supported at a physiological level. It has been emphasized that this increase could reflect altered catecholamine signaling, suggesting that the PPI alteration may be substance-induced [140]. From a dopaminergic perspective, cocaine is known to block dopamine (DA) reuptake pumps, leading to increased levels of free DA [155, 156]. This increase in DA has been associated with a continuous increase in the alerting response [157, 158]. However, most studies evaluate patients during abstinence, where a deficit in DA production has been observed due to tolerance, leading to a decrease in startle response and an increase in PPI among these patients [159, 160]. This increased PPI would lead to enhanced pre-attentional automatic processes that result in greater sensitivity to rewards, further focusing on the pleasurable stimuli associated with the drug [144].

Overall, forty-two studies of the sixty-four reviewed (65.62%) found reduced PPI in patients compared to controls. The absence of differences in the remaining studies can be partially explained by the large proportion of patients under psychopharmacological medication at the time of experimental data collection, that tend to normalize PPI [65, 161, 162]. Additionally, two studies that used pediatric samples found no differences [93, 96], which may be due to the high rate of psychostimulant medicalization at these ages [163]. Furthermore, as mentioned previously, it should be noted that the paradoxical effects of stimulant substance use lead to higher PPI levels in patients compared to controls in certain studies. Finally, it should be noted that other factors such as insomnia [164], motivation [148], and affective processes [51] have not been controlled in the reviewed studies, and could be affecting to PPI magnitude.

In the same vein, differences in experimental parameters can affect the results. Thus, for instance, the higher differences appeared when using an inter-stimulus interval between the prepulse and the pulse of 60 ms [105, 113, 120], and 120 ms [91, 92, 102, 110, 115]. With lower values the occurrence of PPI could be hindered [165], while higher values induce a prepulse facilitation effect [166].

Regarding the potential neurobiological factors related to PPI disruption, the studies reviewed generally point to deficits in the medial prefrontal cortex [100, 109, 116, 117, 122], the corticostriatal-pallidopontine limbic circuit [87, 91, 92, 123, 129, 128], the basal ganglia [84, 106, 115, 129–132], the amygdala [95, 110, 122], the nucleus of the stria terminalis, and the hippocampus [81, 82, 122]. These areas had previously been related to PPI in both animals and humans [41, 44–46]. Additionally, changes in neurotransmission such as hyperactivation of the dopaminergic system [95, 83, 115, 134, 142, 143] and dysfunction of the glutamatergic, GABAergic, and catecholaminergic systems have also been associated with PPI disruption [84, 89, 90, 135, 140].

The reviewed studies have proposed different potential cognitive factors associated with PPI deficit such as re-experiencing and experiential avoidance [85, 111, 113], obsessive-compulsive symptoms severity [123, 126, 128, 130], inability to disengage attention [104], hypervigilance [105], and cognitive deficits related to attention, inhibitory control, planning, verbal memory, or the speed of information processing [84, 100, 108, 117, 119, 137–139]. In fact, experiential avoidance is an important vulnerability factor for anxiety and stress [167–169], that have also been related to PPI disruption [70, 122].

The connection between cognitive performance and PPI disruption has not been fully elucidated. Some studies find an association between PPI levels and performance in tasks assessing sustained attention, working memory, and executive function [170]. In this line, Geyer [26] proposed that PPI deficit could lead to or be predictive of severe deficits in cognitive function. Thus, classical studies proposed that a disruption in PPI would indicate impaired inhibitory filtering, such that irrelevant stimuli could not be correctly filtered, causing a sensory overload, and subsequent failures of higher cognitive functions [56, 171]. However, this classical hypothesis, and therefore the correlative relationship between PPI and cognitive performance, has not been demonstrated, with inconsistent results in a multitude of recent studies that do not find clear neither strong associations between these variables [58, 110, 172, 173].

In addition to the disorders included in this review, deficits in PPI consistently appear in other pathologies such as schizophrenia spectrum disorders [58], autism spectrum [62], neurodevelopmental disorders [63], and schizotypal personality disorder [60, 61]. These disorders share affective components [174, 175], such as stress. This factor has been related to sensorimotor gating deficits through changes on dopaminergic activity. Thus, a higher level of stress produces an increase in dopaminergic activity [176, 177], which has also been associated with PPI disruption [43, 49]. Moreover, this deficit in prepulse inhibition could represent a general inability to suppress irrelevant processes [37], such as intrusive thoughts and repetitive behaviors, which are typical and shared elements among these disorders [21, 68].

Through specific analysis, the group of disorders where this deficit in PPI is most evident is within the schizophrenia spectrum disorders [58], as well as in conditions closely related to the spectrum, such as schizotypal personality disorder [60, 61]. In schizophrenia, PPI has been proposed as a key paradigm for studying the disease, understanding this deficit as an idiosyncratic characteristic of the disorder, while also being proposed as an endophenotype [55–57] and a biomarker [53, 54]. However, when studying individuals at genetic risk for schizophrenia as first-degree relatives, it has been found intact PPI [59]. It is believed that these contradictory findings in the deficit of sensorimotor gating in the prodromal stages of schizophrenia may be due to methodological differences between studies, as PPI would have relevant genetic components and it has been proposed as an endogenous phenotype in pedigree studies [59].

Regarding autism spectrum disorders, a clear decrease in PPI appears to be evident among children and adolescents with these disorders compared to controls. However, the consistency in adults within this spectrum is lower [62]. This difference could be attributed to the fact that it has been demonstrated that PPI for stimulus intervals of 60ms and 120ms increases progressively from 3 years to reach the adult levels from 9 to 10 years of age [178], which would result in a more pronounced deficit among younger individuals within the autism spectrum compared to adults, as they may experience a delay or disorganization in PPI maturation [62].

Finally, in neurodevelopmental disorders, a divergent deficit in PPI emerges when comparing patients to controls across various disorders [63], such as enuresis and childhood-onset fluency disorder [63, 68]. However, in individuals diagnosed with attention-deficit/hyperactivity disorder (ADHD), the deficit in sensorimotor gating is less evident [63]. The fact that this deficit is not as pronounced in ADHD may be attributed to the high rate of medicalization with psychostimulants in this disorder [163], which influence the PPI index, normalizing it [65]. As a result, no differences may be observed when comparing these patients to control groups.

The PPI deficit has been found in most of the eighteen psychopathological conditions that comprise the four groups of neuropsychiatric disorders included in this review. This result is in line with the wide variety of neuropsychiatric disorders in which a sensorimotor gating deficit has been reported in the scientific literature [21, 26, 68], that goes beyond diagnostic boundaries and supports its value as a possible transdiagnostic process [179], and as a translational research measure suitable for clinical practice [47]. Given the evidence that sensory gating measures, such as P50 suppression, have also been found to be altered in a wide range of psychopathological conditions [24, 180–183], it is not surprising that PPI deficit can be proposed as a transdiagnostic mechanism.

From an applied perspective, PPI represents a measure of the integrity of the central nervous system, being a neurobiological operational measure that could reflect the functioning of the dopaminergic system [49, 52]. Considering the transdiagnostic nature of the deficit, and that it could be considered as an index of susceptibility or psychopathological risk, in the future, when the representativeness of the studies is greater and the studies include samples of patients at different stages of the disorders, it could be employed as a marker of state-trait disease. Therefore, PPI could become a screening measure that would facilitate the referral of patients to specialized mental health services, in the same way as processes such as the delay discounting [184, 185], or the N-400 component [186].

Regarding the research domain, PPI could be used as an index of nervous system integrity in clinical trials of new psychotropic drugs, as well as in the study of advanced neuromodulation therapies such as deep brain stimulation, as has already been done successfully in rodents’ [187, 188] and humans’ studies [130, 189, 190]. Additionally, it could be used as a measure to assess therapeutic changes in evidence-based psychological therapies, as has been done with other paradigms such as the P3 component in electroencephalography (e.g., Harris and Hall [191]; Vázquez-Marrufo et al. [192]) or several functional MRI techniques in brain injury rehabilitation (e.g., Rios-Lago et al. [193]; Muñoz-Cespedes et al. [194]).

### Limitations and future perspectives

A limitation of this systematic review is that it included studies covering a wide range of psychopathologies with heterogeneous experimental parameters and designs. Due to this heterogeneity, and considering that many studies did not report effect sizes nor allow accessibility to the original datasets, we were unable to conduct a quantitative synthesis of the reviewed literature that would provide stronger evidence on the transdiagnostic status of PPI deficit. It should also be noted that none of the studies reviewed included control for several factors that affect PPI magnitude, such as insomnia or affective factors (e.g., stress, motivation, etc.).

In the reviewed studies, an effort has been made to integrate the research and applied fields regarding the deficit in PPI. This aligns with the Research Domain Criteria (RDoC), which is interested in the underlying mechanisms of mental disorders by linking cognitive, neural, and behavioral levels of analysis [7, 8]. In this review, the standards of the RDoC framework have been followed by analyzing PPI in different psychopathology groups and attempting to integrate these levels, proposing that the PPI deficit could be a transdiagnostic deficit. However, many studies reviewed did not report conclusions at neurobiological or cognitive levels. Hence, it would be interesting for future studies using this methodological framework to conduct in-depth and multisystem analyses of PPI to empirically verify its transdiagnostic nature in mental disorders, thus confirming its utility in translational research, as has been done with other variables such as aggression proneness [195].

Regarding the usefulness of PPI as a measure for neuropsychiatric disorders, it should be noted that while PPI deficits have been found in several disorders, it can also be normal in some individuals with these disorders and reduced in some healthy individuals. Additionally, PPI is not a stable trait and can be influenced by external factors such as stress, medication, and sleep. Therefore, PPI does not provide additional information on diagnosis or clinical course beyond what can be determined through a standard psychiatric interview or neurological examination [196], but in conjunction with other tools and in a controlled experimental environment, PPI can be valuable in explaining changes and imbalances in clinical conditions [68].

Finally, it is needed to study the different processes that modulate startle response and/or PPI in order to get a better understanding of such measures, as well as to develop future interventions targeting modulatory factors. Thus, it would be important to include in future studies processes such as stress, goal-directed attention or motivation. Moreover, since other reported transdiagnostic processes such as intolerance of uncertainty seem to be a mechanism that maintains psychopathology [16, 197], it would be interesting to study a potential role of the PPI deficit in the genesis and/or maintenance of neuropsychiatric disorders moving from cross-sectional research through longitudinal designs in order to study the course of the disorders.

## Conclusion

In summary, this systematic review represents an integration of the different levels of analysis of a psychopathological process, such as the deficit in the PPI of the startle response. The results have revealed a moderate degree of consistency on PPI deficit in the groups of disorders related to trauma-, stress- and anxiety-related, mood-related, neurocognitive and other disorders such as obsessive-compulsive, tic-related, and substance use disorders, as well as an approach from the transdiagnostic methodology to the study of this phenomenon. The evidence described will allow progress in the understanding of the PPI deficit as a relevant phenomenon in psychopathology, as well as its use as a translational mechanism, thus allowing early detection and intervention in neuropsychiatric disorders.

### Electronic supplementary material

Below is the link to the electronic supplementary material.

Supplementary Material 1

## Data Availability

The data that support the findings of this study are available from the corresponding author upon request.

## References

[1] Newby JM, McKinnon A, Kuyken W, Gilbody S, Dalgleish T (2015). Systematic review and meta-analysis of transdiagnostic psychological treatments for anxiety and depressive disorders in adulthood. Clin Psychol Rev.

[2] Fusar-Poli P, Solmi M, Brondino N, Davies C, Chae C, Politi P, Borgwardt S, Lawrie SM, Parnas J, McGuire P (2019). Transdiagnostic psychiatry: a systematic review. World Psychiatry.

[3] Buckholtz JW, Meyer-Lindenberg A (2012). Psychopathology and the human connectome: toward a transdiagnostic model of risk for mental illness. Neuron.

[4] González Pando D, Cernuda Martínez JA, Alonso Pérez F, Beltrán García P, Aparicio Basauri V (2018). Transdiagnóstico: origen e implicaciones en los cuidados de salud mental. Rev Asoc Esp Neuropsiq.

[5] Sakiris N, Berle D (2019). A systematic review and meta-analysis of the Unified Protocol as a transdiagnostic emotion regulation based intervention. Clin Psychol Rev.

[6] Sanislow CA, Pine DS, Quinn KJ, Kozak MJ, Garvey MA, Heinssen RK, Wang PS, Cuthbert BN (2010). Developing constructs for psychopathology research: research domain criteria. J Abnorm Psychol.

[7] Sanislow CA (2020). RDoC at 10: changing the discourse for psychopathology. World Psychiatry.

[8] Insel TR (2014). The NIMH research domain criteria (RDoC) project: precision medicine for psychiatry. Am J Psychiat.

[9] Sonuga-Barke EJ (2014). What’s up,(R) DoC?’–can identifying core dimensions of early functioning help us understand, and then reduce, developmental risk for mental disorders?. J Child Psychol Psychiatry.

[10] Dalgleish T, Black M, Johnston D, Bevan A (2020). Transdiagnostic approaches to mental health problems: current status and future directions. J Consult Clin Psych.

[11] Mansell W, Harvey A, Watkins ER, Shafran R (2008). Cognitive behavioral processes across psychological disorders: a review of the utility and validity of the transdiagnostic approach. Int J Cogn Ther.

[12] Ellard KK, Bernstein EE, Hearing C, Baek JH, Sylvia LG, Nierenberg AA, Barlow DH, Deckersbach T (2017). Transdiagnostic treatment of bipolar disorder and comorbid anxiety using the Unified Protocol for Emotional Disorders: a pilot feasibility and acceptability trial. J Affect Disord.

[13] Sandín B, Chorot P, Valiente RM (2012). Transdiagnostic: a new frontier in clinical psychology. Rev Psicopatol Psicol Clin.

[14] Levin ME, MacLane C, Daflos S, Seeley JR, Hayes SC, Biglan A, Pistorello J (2014). Examining psychological inflexibility as a transdiagnostic process across psychological disorders. J Contextual Behav Sci.

[15] Harvey AG, Murray G, Chandler RA, Soehner A (2011). Sleep disturbance as transdiagnostic: consideration of neurobiological mechanisms. Clin Psychol Rev.

[16] Rosser BA (2019). Intolerance of uncertainty as a transdiagnostic mechanism of psychological difficulties: a systematic review of evidence pertaining to causality and temporal precedence. Cogn Ther Res.

[17] González M, Ibáñez I, Barrera A (2017). Rumination, worry and negative problem orientation: transdiagnostic processes of anxiety, eating behavior and mood disorders. Acta Colomb Psicol.

[18] González M, Ibáñez I, Rovella A, López M, Padilla L. Perfeccionismo e intolerancia a la incertidumbre: relaciones con variables psicopatológicas. Behav Psychol. 2013;21(1).

[19] Ehring T, Watkins ER (2008). Repetitive negative thinking as a transdiagnostic process. Int J Cogn Ther.

[20] Ellickson-Larew S, Stasik-O’Brien SM, Stanton K, Watson D (2020). Dissociation as a multidimensional transdiagnostic symptom. Psychol Conscious: Theory Res Pract.

[21] Braff DL, Geyer MA, Swerdlow NR (2001). Human studies of prepulse inhibition of startle: normal subjects, patient groups, and pharmacological studies. Psychopharmacology.

[22] Greeson J, Garland EL, Black D. Mindfulness: a transtherapeutic approach for transdiagnostic mental processes. In: Ie A, Ngnoumen CT, Langer EJ, editors. The Wiley Blackwell handbook of mindfulness. Wiley Blackwell; 2014. pp. 533–62. 10.1002/9781118294895.ch28

[23] Oranje B, Geyer MA, Bocker KB, Kenemans JL, Verbaten MN (2006). Prepulse inhibition and P50 suppression: commonalities and dissociations. Psychiatry Res.

[24] Braff DL, Light GA, Swerdlow NR (2007). Prepulse inhibition and P50 suppression are both deficient but not correlated in schizophrenia patients. Biol Psychiatry.

[25] Swerdlow NR, Geyer MA, Hartman PL, Sprock J, Auerbach PP, Cadenhead K, Perry W, Braff DL (1999). Sex differences in sensorimotor gating of the human startle reflex: all smoke?. Psychopharmacology.

[26] Geyer MA (2006). The family of sensorimotor gating disorders: comorbidities or diagnostic overlaps?. Neurotox Res.

[27] Dawson ME, Schell AM, Bohmelt AH. Startle modification: implications for neuroscience, cognitive science, and clinical science. In: Dawson ME, Schell AM, Böhmelt AH, editors. Startle modification. Cambridge University Press; 1999. pp. 6–20.

[28] Carlsen AN, Maslovat D (2019). Startle and the StartReact effect: physiological mechanisms. J Clin Neurophysiol.

[29] Pilz PK, Schnitzler HU (1996). Habituation and sensitization of the acoustic startle response in rats: amplitude, threshold, and latency measures. Neurobiol Learn Mem.

[30] Hoffman HS, Searle JL (1968). Acoustic and temporal factors in the evocation of startle. J Acoust Soc Am.

[31] Graham FK (1975). The more or less startling effects of weak prestimulation. Psychophysiology.

[32] Swerdlow NR. Prepulse Inhibition of Startle in humans and Laboratory Models. In: Squire LR, editor. Encyclopedia of Neuroscience. Academic Press; 2009. pp. 947–55. 10.1016/B978-008045046-9.01938-0

[33] Blumenthal TD, Cuthbert BN, Filion DL, Hackley S, Lipp OV, Van Boxtel A (2005). Committee report: guidelines for human startle eyeblink electromyographic studies. Psychophysiology.

[34] Swerdlow NR (2013). Update: studies of prepulse inhibition of startle, with particular relevance to the pathophysiology or treatment of Tourette Syndrome. Neurosci Biobehav R.

[35] Swerdlow NR, Braff DL, Taaid N, Geyer MA (1994). Assessing the validity of an animal model of deficient sensorimotor gating in schizophrenic patients. Arch Gen Psychiatry.

[36] Geyer MA, Krebs-Thomson K, Braff DL, Swerdlow NR (2001). Pharmacological studies of prepulse inhibition models of sensorimotor gating deficits in schizophrenia: a decade in review. Psychopharmacology.

[37] Swerdlow NR, Caine SB, Braff DL, Geyer MA (1992). The neural substrates of sensorimotor gating of the startle reflex: a review of recent findings and their implications. J Psychopharmacol.

[38] Holstein DH, Vollenweider FX, Geyer MA, Csomor PA, Belser N, Eich D (2013). Sensory and sensorimotor gating in adult attention-deficit/hyperactivity disorder (ADHD). Psychiatry Res.

[39] Blumenthal TD, Reynolds JZ, Spence TE (2015). Support for the interruption and protection hypotheses of prepulse inhibition of startle: evidence from a modified attention network test. Psychophysiology.

[40] Fulcher N, Azzopardi E, De Oliveira C, Hudson R, Schormans AL, Zaman T, Allman BL, Laviolette SR, Schmid S (2020). Deciphering midbrain mechanisms underlying prepulse inhibition of startle. Prog Neurobiol.

[41] Swerdlow NR, Geyer MA, Braff DL (2001). Neural circuit regulation of prepulse inhibition of startle in the rat: current knowledge and future challenges. Psychopharmacology.

[42] Schmajuk NA, Larrauri JA, De la Casa LG, Levin ED (2009). Attenuation of auditory startle and prepulse inhibition by unexpected changes in ambient illumination through dopaminergic mechanisms. Behav Brain Res.

[43] Vargas JP, Díaz E, Portavella M, López JC (2016). Animal models of maladaptive traits: disorders in sensorimotor gating and attentional quantifiable responses as possible endophenotypes. Front Psychol.

[44] Hazlett EA, Buchsbaum MS, Zhang J, Newmark RE, Glanton CF, Zelmanova Y, Haznedar MM, Chu KW, Nenadic I, Kemether EM, Tang CY, New AS, Siever LJ (2008). Frontal–striatal–thalamic mediodorsal nucleus dysfunction in schizophrenia-spectrum patients during sensorimotor gating. NeuroImage.

[45] Li L, Du Y, Li N, Wu X, Wu Y (2009). Top–down modulation of prepulse inhibition of the startle reflex in humans and rats. Neurosci Biobehav Rev.

[46] Miller EJ, Saint Marie LR, Breier MR, Swerdlow NR (2010). Pathways from the ventral hippocampus and caudal amygdala to forebrain regions that regulate sensorimotor gating in the rat. Neuroscience.

[47] Takahashi H, Hashimoto R, Iwase M, Ishii R, Kamio Y, Takeda M (2011). Prepulse inhibition of startle response: recent advances in human studies of psychiatric disease. Clin Psychopharmacol Neurosci.

[48] Powell SB, Weber M, Geyer MA. Genetic models of sensorimotor gating: relevance to neuropsychiatric disorders. Behav Neurogenet. 2011:251–318. 10.1007/7854_2011_195 PMC3357439 22367921

[49] Swerdlow NR, Braff DL, Geyer MA (2016). Sensorimotor gating of the startle reflex: what we said 25 years ago, what has happened since then, and what comes next. J Psychopharmacol.

[50] Arenas MC, Caballero-Reinaldo C, Navarro-Frances CI, Manzanedo C (2017). Effects of cocaine on prepulse inhibition of the startle response. Rev Neurol.

[51] De la Casa LG, Mena A, Puentes A. Startle response and prepulse inhibition modulation by positive-and negative-induced affect. Int J Psychophysiol. 2014;91(2), 73–79. 2014. 10.1016/j.ijpsycho.2013.10.017 10.1016/j.ijpsycho.2013.10.017 24188916

[52] Pujante-Gil S, Manzanedo C, Arenas MC (2021). Effect of stress on prepulse inhibition: a systematic review. Rev Neurol.

[53] Light GA, Swerdlow NR, Rissling AJ, Radant A, Sugar CA, Sprock J, Pela M, Geyer MA, Braff DL (2012). Characterization of neurophysiologic and neurocognitive biomarkers for use in genomic and clinical outcome studies of schizophrenia. PLoS ONE.

[54] Mena A, Ruiz-Salas JC, Puentes A, Dorado I, Ruiz-Veguilla M, De la Casa LG (2016). Reduced prepulse inhibition as a biomarker of schizophrenia. Front Behav Neurosci.

[55] Braff DL, Stone C, Callaway E, Geyer M, Glick I, Bali L (1978). Prestimulus effects on human startle reflex in normals and schizophrenics. Psychophysiology.

[56] Geyer MA, Braff DL (1987). Startle habituation and sensorimotor gating in schizophrenia and related animal models. Schizophr Bull.

[57] Grillon C, Ameli R, Charney DS, Krystal J, Braff D (1992). Startle gating deficits occur across prepulse intensities in schizophrenic patients. Biol Psychiatry.

[58] San-Martin R, Castro LA, Menezes PR, Fraga FJ, Simões PW, Salum C (2020). Meta-analysis of sensorimotor gating deficits in patients with schizophrenia evaluated by prepulse inhibition test. Schizophr Bull.

[59] Li W, Mao Z, Bo Q, Sun Y, Wang Z, Wang C (2021). Prepulse inhibition in first-degree relatives of schizophrenia patients: a systematic review. Early Interv Psychiatry.

[60] Cadenhead KS, Geyer MA, Braff DL (1993). Impaired startle prepulse inhibition and habituation in patients with schizotypal personality disorder. Am J Psychiat.

[61] Wan L, Thomas Z, Pisipati S, Jarvis SP, Boutros NN (2017). Inhibitory deficits in prepulse inhibition, sensory gating, and antisaccade eye movement in schizotypy. Int J Psychophysiol.

[62] Cheng CH, Chan PY, Hsu SC, Liu CY (2018). Meta-analysis of sensorimotor gating in patients with autism spectrum disorders. Psychiatry Res.

[63] Schulz SE, Luszawski M, Hannah KE, Stevenson RA (2023). Sensory gating in Neurodevelopmental Disorders: a scoping review. J Clin Child Adolesc Psychol.

[64] Ashare RL, Hawk LW, Shiels K, Rhodes JD, Pelham WE, Waxmonsky JG (2010). Methylphenidate enhances prepulse inhibition during processing of task-relevant stimuli in attention‐deficit/hyperactivity disorder. Psychophysiology.

[65] Hawk LW, Yartz AR, Pelham WE, Lock TM (2003). The effects of methylphenidate on prepulse inhibition during attended and ignored prestimuli among boys with attention-deficit hyperactivity disorder. Psychopharmacology.

[66] Shibagaki M, Yamanaka T (1990). Attention of hyperactive preschool children—electrodermal activity during auditory stimulation. Percept Mot Skills.

[67] American Psychiatric Association (2013). DSM-5 Task Force Diagnostic and statistical manual of mental disorders: DSM-5.

[68] Kohl S, Heekeren K, Klosterkötter J, Kuhn J (2013). Prepulse inhibition in psychiatric disorders–apart from schizophrenia. J Psychiatr Res.

[69] Valsamis B, Schmid S (2011). Habituation and prepulse inhibition of acoustic startle in rodents. J Vis Exp.

[70] García Sánchez F, Martínez Gras I, Rodríguez Jiménez R, Rubio G. Inhibición prepulso del reflejo de la respuesta de sobresalto en los trastornos neuropsiquiátricos. Rev Neurol. 2011:422–32. 21948013

[71] Ahmari SE, Risbrough VB, Geyer MA, Simpson HB (2016). Prepulse inhibition deficits in obsessive-compulsive disorder are more pronounced in females. Neuropsychopharmacology.

[72] Schall U, Schön A, Zerbin D, Eggers C, Oades RD (1996). Event-related potentials during an auditory discrimination with prepulse inhibition in patients with schizophrenia, obsessive-compulsive disorder and healthy subjects. Int J Neurosci.

[73] Swerdlow NR, Bongiovanni MJ, Tochen L, Shoemaker JM (2006). Separable noradrenergic and dopaminergic regulation of prepulse inhibition in rats: implications for predictive validity and Tourette Syndrome. Psychopharmacology.

[74] Schilke ED, Tremolizzo L, Appollonio I, Ferrarese C (2022). Tics: neurological disorders determined by a deficit in sensorimotor gating processes. Neurol Sci.

[75] Alsuhaibani R, Smith DC, Lowrie R, Aljhani S, Paudyal V (2021). Scope, quality and inclusivity of international clinical guidelines on mental health and substance abuse in relation to dual diagnosis, social and community outcomes: a systematic review. BMC Psychiatry.

[76] Page MJ, McKenzie JE, Bossuyt PM, Boutron I, Hoffmann TC, Mulrow CD, Shamseer L, Tetzlaff JM, Akl EA, Brennan SE, Chou R (2021). The PRISMA 2020 statement: an updated guideline for reporting systematic reviews. Int J Surg.

[77] Higgins JP, Green S. Cochrane handbook for systematic reviews of interventions 5.1.0. The Cochrane Collaboration. 2011;2011.

[78] Petticrew M (2015). Time to rethink the systematic review catechism? Moving from ‘what works’ to ‘what happens’. Syst Rev.

[79] Wells GA, Shea B, O’Connell D, Peterson J, Welch V, Losos M, et al. The Newcastle-Ottawa Scale (NOS) for assessing the quality of nonrandomised studies in meta-analyses. The Ottawa Hospital Research Institute; 2016. http://www.ohri.ca/programs/clinical_epidemiology/oxford.asp

[80] Wells GA, Shea B, O’Connell D, Peterson J, Welch V, Losos M, Tugwell P (2014). The Newcastle-Ottawa Scale (NOS) for assessing the quality of nonrandomised studies in meta-analyses. BMC Med Res Methodol.

[81] Grillon C, Morgan CA, Davis M, Southwick SM (1998). Effects of experimental context and explicit threat cues on acoustic startle in Vietnam veterans with posttraumatic stress disorder. Biol Psychiatry.

[82] Grillon C, Morgan CA, Davis M, Southwick SM (1998). Effect of darkness on acoustic startle in Vietnam veterans with PTSD. Am J Psychiat.

[83] De la Casa LG, Mena A, Ruiz-Salas JC (2016). Effect of stress and attention on startle response and prepulse inhibition. Physiol Behav.

[84] Swerdlow NR, Paulsen J, Braff DL, Butters N, Geyer MA, Swenson MR (1995). Impaired prepulse inhibition of acoustic and tactile startle response in patients with Huntington’s disease. J Neurol Neurosurg Psychiatry.

[85] Grillon C, Morgan CA, Southwick SM, Davis M, Charney DS (1996). Baseline startle amplitude and prepulse inhibition in Vietnam veterans with posttraumatic stress disorder. Psychiatry Res.

[86] Grillon C, Dierker L, Merikangas KR (1997). Startle modulation in children at risk for anxiety disorders and/or alcoholism. J Am Acad Child Adolesc Psychiatr.

[87] Perry W, Minassian A, Feifel D, Braff DL (2001). Sensorimotor gating deficits in bipolar disorder patients with acute psychotic mania. Biol Psychiatry.

[88] Ludewig S, Ludewig K, Geyer MA, Hell D, Vollenweider FX (2002). Prepulse inhibition deficits in patients with panic disorder. Depress Anxiety.

[89] Muñoz E, Cervera A, Valls-Solé J (2003). Neurophysiological study of facial chorea in patients with Huntington’s disease. Clin Neurophysiol.

[90] Hejl AM, Glenthøj B, Mackeprang T, Hemmingsen R, Waldemar G (2004). Prepulse inhibition in patients with Alzheimer’s disease. Neurobiol Aging.

[91] Perry W, Minassian A, Feifel D (2004). Prepulse inhibition in patients with non-psychotic major depressive disorder. J Affect Disord.

[92] Perriol MP, Dujardin K, Derambure P, Marcq A, Bourriez JL, Laureau E, Pasquier F, Defebvre L, Destee A (2005). Disturbance of sensory filtering in dementia with Lewy bodies: comparison with Parkinson’s disease dementia and Alzheimer’s disease. J Neurol Neurosurg Psychiatry.

[93] Rich BA, Vinton D, Grillon C, Bhangoo RK, Leibenluft E (2005). An investigation of prepulse inhibition in pediatric bipolar disorder. Bipolar Disord.

[94] Barrett SL, Kelly C, Watson DR, Bell R, King DJ (2005). Normal levels of prepulse inhibition in the euthymic phase of bipolar disorder. Psychol Med.

[95] Ludewig S, Geyer MA, Ramseier M, Vollenweider FX, Rechsteiner E, Cattapan-Ludewig K (2005). Information-processing deficits and cognitive dysfunction in panic disorder. J Psychiatry Neurosci.

[96] Lipschitz DS, Mayes LM, Rasmusson AM, Anyan W, Billingslea E, Gueorguieva R, Southwick SM (2005). Baseline and modulated acoustic startle responses in adolescent girls with posttraumatic stress disorder. J Am Acad Child Adolesc Psychiatr.

[97] Quednow BB, Westheide J, Kühn KU, Werner P, Maier W, Hawellek B, Wagner M (2006). Normal prepulse inhibition and habituation of acoustic startle response in suicidal depressive patients without psychotic symptoms. J Affect Disord.

[98] Ueki A, Goto K, Sato N, Iso H, Morita Y (2006). Prepulse inhibition of acoustic startle response in mild cognitive impairment and mild dementia of Alzheimer type. Psychiatry Clin Neurosci.

[99] Carroll CA, Vohs JL, O’donnell BF, Shekhar A, Hetrick WP (2007). Sensorimotor gating in manic and mixed episode bipolar disorder. Bipolar Disord.

[100] Giakoumaki SG, Roussos P, Rogdaki M, Karli C, Bitsios P, Frangou S (2007). Evidence of disrupted prepulse inhibition in unaffected siblings of bipolar disorder patients. Biol Psychiatry.

[101] Duley AR, Hillman CH, Coombes S, Janelle CM (2007). Sensorimotor gating and anxiety: prepulse inhibition following acute exercise. Int J Psychophysiol.

[102] Gogos A, van den Buuse M, Rossell S (2009). Gender differences in prepulse inhibition (PPI) in bipolar disorder: men have reduced PPI, women have increased PPI. Int J Neuropsychopharmacol.

[103] Holstein DH, Vollenweider FX, Jäncke L, Schopper C, Csomor PA (2010). P50 suppression, prepulse inhibition, and startle reactivity in the same patient cohort suffering from posttraumatic stress disorder. J Affect Disord.

[104] McMillan KA, Asmundson GJ, Zvolensky MJ, Carleton RN (2012). Startle response and anxiety sensitivity: subcortical indices of physiologic arousal and fear responding. Emotion.

[105] Vrana SR, Calhoun PS, McClernon FJ, Dennis MF, Lee ST, Beckham JC (2013). Effects of smoking on the acoustic startle response and prepulse inhibition in smokers with and without posttraumatic stress disorder. Psychopharmacology.

[106] Zoetmulder M, Biernat HB, Nikolic M, Korbo L, Jennum PJ (2014). Sensorimotor gating deficits in multiple system atrophy: comparison with Parkinson’s disease and idiopathic REM sleep behavior disorder. Parkinsonism Relat Disord.

[107] Ivleva EI, Moates AF, Hamm JP, Bernstein IH, O’Neill HB, Cole D, Clementz BA, Thaker GK, Tamminga CA (2014). Smooth pursuit eye movement, prepulse inhibition, and auditory paired stimuli processing endophenotypes across the schizophrenia-bipolar disorder psychosis dimension. Schizophr Bull.

[108] Comasco E, Hellgren C, Olivier J, Skalkidou A, Poromaa IS (2015). Supraphysiological hormonal status, anxiety disorders, and COMT Val/Val genotype are associated with reduced sensorimotor gating in women. Psychoneuroendocrinology.

[109] Vrana SR, Calhoun PS, Dennis MF, Kirby AC, Beckham JC (2015). Acoustic startle and prepulse inhibition predict smoking lapse in posttraumatic stress disorder. J Psychopharmacol.

[110] Sánchez-Morla EM, Mateo J, Aparicio A, García‐Jiménez M, Jiménez E, Santos JL (2016). Prepulse inhibition in euthymic bipolar disorder patients in comparison with control subjects. Acta Psychiatr Scand.

[111] Pineles SL, Blumenthal TD, Curreri AJ, Nillni YI, Putnam KM, Resick PA, Rasmusson AM, Orr SP (2016). Prepulse inhibition deficits in women with PTSD. Psychophysiology.

[112] Comasco E, Gulinello M, Hellgren C, Skalkidou A, Sylven S, Sundström-Poromaa I (2016). Sleep duration, depression, and oxytocinergic genotype influence prepulse inhibition of the startle reflex in postpartum women. Eur Neuropsychopharmacol.

[113] Echiverri-Cohen AM, Zoellner LA, Ho W, Husain J (2016). An analysis of inhibitory functioning in individuals with chronic posttraumatic stress disorder. J Anxiety Disord.

[114] Matsuo J, Ota M, Hidese S, Hori H, Teraishi T, Ishida I, Hiraishi M, Kunugi H (2017). Sexually dimorphic deficits of prepulse inhibition in patients with major depressive disorder and their relationship to symptoms: a large single ethnicity study. J Affect Disord.

[115] Millian-Morell L, Lopez-Alburquerque T, Rodriguez-Rodriguez A, Gomez-Nieto R, Carro J, Meilan JJ, Martinez-Sanchez F, Sancho C, Lopez DE (2018). Relations between sensorimotor integration and speech disorders in Parkinson’s disease. Curr Alzheimer Res.

[116] Meteran H, Vindbjerg E, Uldall SW, Glenthøj B, Carlsson J, Oranje B (2019). Startle habituation, sensory, and sensorimotor gating in trauma-affected refugees with posttraumatic stress disorder. Psychol Med.

[117] Bo Q, Mao Z, Tian Q, Wen Y, Dong F, Li X, Wang Z, Ma X, Wang C (2018). Deficits of perceived spatial separation-induced prepulse inhibition in patients with bipolar disorder compared to healthy controls. J Affect Disord.

[118] Matsuo J, Ota M, Hidese S, Teraishi T, Hori H, Ishida I, Hiraishi M, Kunugi H (2018). Sensorimotor gating in depressed and euthymic patients with bipolar disorder: analysis on prepulse inhibition of acoustic startle response stratified by gender and state. Front Psychiatry.

[119] Massa N, Owens AV, Harmon W, Bhattacharya A, Ivleva EI, Keedy S, Sweeney JA, Pearlson GD, Keshavan MS, Tamminga CA, Clementz BA (2020). Relationship of prolonged acoustic startle latency to diagnosis and biotype in the bipolar-schizophrenia network on intermediate phenotypes (B–SNIP) cohort. Schizophr Res.

[120] Storozheva ZI, Akhapkin RV, Bolotina OV, Korendrukhina A, Novototsky-Vlasov VY, Shcherbakova IV, Kirenskaya AV (2021). Sensorimotor and sensory gating in depression, anxiety, and their comorbidity. World J Biol Psychiatry.

[121] San-Martin R, Zimiani MI, de Ávila MA, Shuhama R, Del-Ben CM, Menezes PR, Fraga FJ, Salum C (2022). Early Schizophrenia and bipolar disorder patients display reduced neural prepulse inhibition. Brain Sci.

[122] Acheson DT, Baker DG, Nievergelt CM, Yurgil KA, Geyer MA, Risbrough VB (2022). Prospective longitudinal assessment of sensorimotor gating as a risk/resiliency factor for posttraumatic stress disorder. Neuropsychopharmacology.

[123] Swerdlow NR, Benbow CH, Zisook S, Geyer MA, Braff DL (1993). A preliminary assessment of sensorimotor gating in patients with obsessive compulsive disorder. Biol Psychiatry.

[124] Castellanos FX, Fine EJ, Kaysen D, Marsh WL, Rapoport JL, Hallett M (1996). Sensorimotor gating in boys with Tourette’s syndrome and ADHD: preliminary results. Biol Psychiatry.

[125] Swerdlow NR, Karban B, Ploum Y, Sharp R, Geyer MA, Eastvold A (2001). Tactile prepuff inhibition of startle in children with Tourette’s syndrome: in search of an “fMRI-friendly” startle paradigm. Biol Psychiatry.

[126] Hoenig K, Hochrein A, Quednow BB, Maier W, Wagner M (2005). Impaired prepulse inhibition of acoustic startle in obsessive-compulsive disorder. Biol Psychiatry.

[127] de Leeuw AS, Oranje B, van Megen HJ, Kemner C, Westenberg HG (2010). Sensory gating and sensorimotor gating in medication-free obsessive-compulsive disorder patients. Int Clin Psychopharmacol.

[128] Ahmari SE, Risbrough VB, Geyer MA, Simpson HB (2012). Impaired sensorimotor gating in unmedicated adults with obsessive–compulsive disorder. Neuropsychopharmacology.

[129] Buse J, Beste C, Herrmann E, Roessner V (2016). Neural correlates of altered sensorimotor gating in boys with Tourette Syndrome: a combined EMG/fMRI study. World J Biol Psychiatry.

[130] Kohl S, Gruendler TO, Huys D, Sildatke E, Dembek TA, Hellmich M, Vorderwulbecke M, Timmermann L, Ahmari SE, Klosterkoetter J, Jessen F (2015). Effects of deep brain stimulation on prepulse inhibition in obsessive-compulsive disorder. Transl Psychiatr.

[131] Zebardast N, Crowley MJ, Bloch MH, Mayes LC, Vander Wyk B, Leckman JF, Pelphrey KA, Swain JE (2013). Brain mechanisms for prepulse inhibition in adults with Tourette syndrome: initial findings. Psychiatry Res.

[132] Pittenger C, Adams TG, Gallezot JD, Crowley MJ, Nabulsi N, Ropchan J, Gao H, Kichuk SA, Simpson R, Billingslea E, Hannestad J (2016). OCD is associated with an altered association between sensorimotor gating and cortical and subcortical 5-HT1b receptor binding. J Affect Disord.

[133] Steinman SA, Ahmari SE, Choo T, Kimeldorf MB, Feit R, Loh S, Risbrough V, Geyer MA, Steinglass JE, Wall M, Schneier FR (2016). Prepulse inhibition deficits only in females with obsessive–compulsive disorder. Depress Anxiety.

[134] Efferen TR, Duncan EJ, Szilagyi S, Chakravorty S, Adams JU, Gonzenbach S, Angrist B, Butler PD, Rotrosen J (2000). Diminished acoustic startle in chronic cocaine users. Neuropsychopharmacology.

[135] Quednow BB, Kühn KU, Hoenig K, Maier W, Wagner M (2004). Prepulse inhibition and habituation of acoustic startle response in male MDMA (‘ecstasy’) users, cannabis users, and healthy controls. Neuropsychopharmacology.

[136] Heekeren K, Daumann J, Geyer MA, Gouzoulis-Mayfrank E (2004). Plasticity of the acoustic startle reflex in currently abstinent ecstasy (MDMA) users. Psychopharmacology.

[137] Kedzior KK, Martin-Iverson MT (2006). Chronic cannabis use is associated with attention-modulated reduction in prepulse inhibition of the startle reflex in healthy humans. J Psychopharmacol.

[138] Kedzior KK, Martin-Iverson MT (2007). Attention-dependent reduction in prepulse inhibition of the startle reflex in cannabis users and schizophrenia patients—a pilot study. Eur J Pharmacol.

[139] Mathias CW, Blumenthal TD, Dawes MA, Liguori A, Richard DM, Bray B, Tong W, Dougherty DM (2011). Failure to sustain prepulse inhibition in adolescent marijuana users. Drug Alcohol Depend.

[140] Preller KH, Ingold N, Hulka LM, Vonmoos M, Jenni D, Baumgartner MR, Vollenweider FX, Quednow BB (2013). Increased sensorimotor gating in recreational and dependent cocaine users is modulated by craving and attention-deficit/hyperactivity disorder symptoms. Biol Psychiatry.

[141] Winton-Brown T, Kumari V, Windler F, Moscoso A, Stone J, Kapur S, McGuire P (2015). Sensorimotor gating, cannabis use and the risk of psychosis. Schizophr Res.

[142] Morales-Muñoz I, Jurado-Barba R, Caballero M, Rodríguez-Jiménez R, Jiménez-Arriero M, Fernández-Guinea S, Rubio G (2015). Cannabis abuse effects on prepulse inhibition in patients with first episode psychosis in schizophrenia. J Neuropsychiatry Clin Neurosci.

[143] Gil-Miravet I, Fuertes-Saiz A, Benito A, Almodóvar I, Ochoa E, Haro G (2021). Prepulse inhibition in cocaine addiction and dual pathologies. Brain Sci.

[144] Echeverria I, Benito A, Fuertes-Saiz A, Graña ML, Aleixandre I, Haro G (2021). Cocaine increases Sensorimotor Gating and is related to psychopathy. J Dual Diagn.

[145] Milner CE, Cuthbert BP, Kertesz RS, Cote KA (2009). Sensory gating impairments in poor sleepers during presleep wakefulness. NeuroReport.

[146] Fried EI, van Borkulo CD, Cramer AO, Boschloo L, Schoevers RA, Borsboom D (2017). Mental disorders as networks of problems: a review of recent insights. Soc Psychiatry Psychiatr Epidemiol.

[147] Mennin DS, Holaway RM, Fresco DM, Moore MT, Heimberg RG (2007). Delineating components of emotion and its dysregulation in anxiety and mood psychopathology. Behav Therapy.

[148] Ashare RL, Hawk LW, Mazzullo RJ (2007). Motivated attention: incentive effects on attentional modification of prepulse inhibition. Psychophysiology.

[149] Archer T, Kostrzewa RM, Beninger RJ, Palomo T (2011). Staging neurodegenerative disorders: structural, regional, biomarker, and functional progressions. Neurotox Res.

[150] Li M, Long C, Yang L. Hippocampal-prefrontal circuit and disrupted functional connectivity in psychiatric and neurodegenerative disorders. Biomed Res Int. 2015;2015. 10.1155/2015/810548 10.1155/2015/810548 PMC4396015 25918722

[151] Xu P, Chen A, Li Y, Xing X, Lu H (2019). Medial prefrontal cortex in neurological diseases. Physiol Genomics.

[152] Schneider M, Koch M (2002). The cannabinoid agonist WIN 55,212-2 reduces sensorimotor gating and recognition memory in rats. Behav Pharmacol.

[153] Schneider M, Drews E, Koch M (2005). Behavioral effects in adult rats of chronic prepubertal treatment with the cannabinoid receptor agonist WIN 55,212-2. Behav Pharmacol.

[154] Gururajan A, Taylor DA, Malone DT (2011). Effect of cannabidiol in a MK-801-rodent model of aspects of schizophrenia. Behav Brain Res.

[155] Koob GF, Volkow ND (2010). Neurocircuitry of addiction. Neuropsychopharmacology.

[156] Volkow ND, Wang GJ, Fowler JS, Tomasi D, Telang F (2011). Addiction: beyond dopamine reward circuitry. Proc Natl Acad Sci.

[157] Kehne JH, Sorenson CA (1978). The effects of pimozide and phenoxybenzamine pretreatments on amphetamine and apomorphine potentiation of the acoustic startle response in rats. Psychopharmacology.

[158] Martinez ZA, Ellison GD, Geyer MA, Swerdlow NR (1999). Effects of sustained cocaine exposure on sensorimotor gating of startle in rats. Psychopharmacology.

[159] Parsons LH, Smith AD, Justice JB (1991). Basal extracellular dopamine is decreased in the rat nucleus accumbens during abstinence from chronic cocaine. Synapse.

[160] Kuhar MJ, Pilotte NS (1996). Neurochemical changes in cocaine withdrawal. Trends Pharmacol Sci.

[161] Ornitz EM, Hanna GL, de Traversay J (1992). Prestimulation-induced startle modulation in attention‐deficit hyperactivity disorder and nocturnal enuresis. Psychophysiology.

[162] Swerdlow NR, Van Bergeijk DP, Bergsma F, Weber E, Talledo J (2009). The effects of memantine on prepulse inhibition. Neuropsychopharmacology.

[163] Graf WD, Miller G, Nagel SK (2014). Addressing the problem of ADHD medication as neuroenhancements. Expert Rev Neurother.

[164] Frau R, Orru M, Puligheddu M, Gessa GL, Mereu G, Marrosu F, Bortolato M (2008). Sleep deprivation disrupts prepulse inhibition of the startle reflex: reversal by antipsychotic drugs. Int J Neuropsychopharmacol.

[165] Reijmers LG, Peeters BW (1994). Effects of acoustic prepulses on the startle reflex in rats: a parametric analysis. Brain Res.

[166] Graham FK, Murray GM (1977). Discordant effects of weak prestimulation on magnitude and latency of the reflex blink. Physiol Psychol.

[167] Berman NC, Wheaton MG, McGrath P, Abramowitz JS (2010). Predicting anxiety: the role of experiential avoidance and anxiety sensitivity. J Anxiety Disord.

[168] Bardeen JR, Fergus TA, Orcutt HK (2014). The moderating role of experiential avoidance in the prospective relationship between anxiety sensitivity and anxiety. Cogn Ther Res.

[169] Bardeen JR, Fergus TA (2016). The interactive effect of cognitive fusion and experiential avoidance on anxiety, depression, stress and posttraumatic stress symptoms. J Contextual Behav Sci.

[170] Giakoumaki SG (2012). Cognitive and prepulse inhibition deficits in psychometrically high schizotypal subjects in the general population: relevance to schizophrenia research. J Int Neuropsychol Soc.

[171] Braff DL, Geyer MA (1990). Sensorimotor gating and schizophrenia: human and animal model studies. Arch Gen Psychiatry.

[172] Swerdlow NR, Light GA (2018). Sensorimotor gating deficits in schizophrenia: advancing our understanding of the phenotype, its neural circuitry and genetic substrates. Schizophr Res.

[173] Togay B, Çıkrıkçılı U, Bayraktaroglu Z, Uslu A, Noyan H, Üçok A (2020). Lower prepulse inhibition in clinical high-risk groups but not in familial risk groups for psychosis compared with healthy controls. Early Interv Psychiatry.

[174] Goel N, Bale TL (2009). Examining the intersection of sex and stress in modelling neuropsychiatric disorders. J Neuroendocrinol.

[175] Musazzi L, Tornese P, Sala N, Popoli M (2018). What acute stress protocols can tell us about PTSD and stress-related neuropsychiatric disorders. Front Pharmacol.

[176] Koob GF, Buck CL, Cohen A, Edwards S, Park PE, Schlosburg JE, Schmeichel B, Vendruscolo LF, Wade CL, Whitfield TW, George O (2014). Addiction as a stress surfeit disorder. Neuropharmacology.

[177] Mizrahi R, Kenk M, Suridjan I, Boileau I, George TP, McKenzie K, Wilson AA, Houle S, Rusjan P (2014). Stress-induced dopamine response in subjects at clinical high risk for schizophrenia with and without concurrent cannabis use. Neuropsychopharmacology.

[178] Gebhardt J, Schulz-Juergensen S, Eggert P (2012). Maturation of prepulse inhibition (PPI) in childhood. Psychophysiology.

[179] Schulz S, Hannah K, Stevenson R (2022). Sensory gating in Neurodevelopmental Disorders: a scoping review. OSF.

[180] Clementz BA, Geyer MA, Braff DL (1997). P50 suppression among schizophrenia and normal comparison subjects: a methodological analysis. Biol Psychiatry.

[181] Olincy A, Martin L (2005). Diminished suppression of the P50 auditory evoked potential in bipolar disorder subjects with a history of psychosis. Am J Psychiat.

[182] Karl A, Malta LS, Maercker A (2006). Meta-analytic review of event-related potential studies in post-traumatic stress disorder. Biol Psychol.

[183] Nanbu M, Kurayama T, Nakazawa K, Matsuzawa D, Komiya Z, Haraguchi T, Ogura H, Hashimoto T, Yoshida S, Iyo M, Shimizu E (2010). Impaired P50 suppression in fear extinction in obsessive–compulsive disorder. Prog Neuro-Psychopharmacol Biol Psychiatry.

[184] Amlung M, Marsden E, Holshausen K, Morris V, Patel H, Vedelago L, Naish KR, Reed DD, McCabe RE (2019). Delay discounting as a transdiagnostic process in psychiatric disorders: a meta-analysis. JAMA psychiatry.

[185] Chen Z, Becker B, Qin P, Lei W, Chen J, Liu P, Lin T, Zhang C, Zhang R, Wang M, Xu T (2021). Neural networks during delay discounting as trans-disease marker: a meta-analytical review. J Psychiatr Res.

[186] Siddiqui SV, Nizamie SH, Siddiqui MA, Jahan M, Garg S, Tikka SK, Shreekantiah U (2021). Evaluation of N-400 evoked response potential in schizophrenia: an endophenotype or a disease marker?. Psychiatry Res.

[187] Bikovsky L, Hadar R, Soto-Montenegro ML, Klein J, Weiner I, Desco M, Pascau J, Winter C, Hamani C (2016). Deep brain stimulation improves behavior and modulates neural circuits in a rodent model of schizophrenia. Exp Neurol.

[188] Elle T, Alam M, Voigt C, Krauss JK, John N, Schwabe K (2020). Deep brain stimulation of the thalamic centromedian-parafascicular nucleus improves behavioural and neuronal traits in a rat model of Tourette. Behav Brain Res.

[189] Gee L, Smith H, De La Cruz P, Campbell J, Fama C, Haller J, Ramirez-Zamora A, Durphy J, Hanspal E, Molho E, Barba A (2015). The influence of bilateral subthalamic nucleus deep brain stimulation on impulsivity and prepulse inhibition in Parkinson’s disease patients. Stereotact Funct Neurosurg.

[190] Schleyken S, Baldermann J, Huys D, Franklin J, Visser-Vandewalle V, Kuhn J, Kohl S (2020). Deep brain stimulation and sensorimotor gating in tourette syndrome and obsessive-compulsive disorder. J Psychiatr Res.

[191] Harris DP, Hall JW (1990). Feasibility of auditory event-related potential measurement in brain injury rehabilitation. Ear Hear.

[192] Vázquez-Marrufo M, González-Rosa JJ, Galvao-Carmona A, Hidalgo-Muñoz A, Borges M, Peña JL, Izquierdo G (2013). Retest reliability of individual p3 topography assessed by high density electroencephalography. PLoS ONE.

[193] Rios-Lago M, Paul-Laprediza N, Munoz-Cespedes JM, Maestu F, Alvarez-Linera J, Ortiz T (2004). Functional neuroimaging applied to the study of neuropsychological rehabilitation. Rev Neurol.

[194] Muñoz-Cespedes JM, Rios-Lago M, Paul N, Maestu F (2005). Functional neuroimaging studies of cognitive recovery after acquired brain damage in adults. Neuropsychol Rev.

[195] Verona E, Bresin K (2015). Aggression proneness: transdiagnostic processes involving negative valence and cognitive systems. Int J Psychophysiol.

[196] Swerdlow NR, Weber M, Qu Y, Light GA, Braff DL (2008). Realistic expectations of prepulse inhibition in translational models for schizophrenia research. Psychopharmacology.

[197] Carleton RN (2016). Into the unknown: a review and synthesis of contemporary models involving uncertainty. J Anxiety Disord.

